# Endobronchial Coils for Severe Emphysema Are Effective Up to 12 Months following Treatment: Medium Term and Cross-Over Results from a Randomised Controlled Trial

**DOI:** 10.1371/journal.pone.0122656

**Published:** 2015-04-08

**Authors:** Zaid Zoumot, Samuel V. Kemp, Suveer Singh, Stephen R. Bicknell, William H. McNulty, Nicholas S. Hopkinson, Ewen T. Ross, Pallav L. Shah

**Affiliations:** 1 The NIHR Respiratory Biomedical Research Unit at the Royal Brompton and Harefield NHS Foundation Trust and Imperial College, London, United Kingdom; 2 Chelsea & Westminster Hospital NHS Foundation Trust, London, United Kingdom; 3 Respiratory and Critical Care Institute, Cleveland Clinic Abu Dhabi, Abu Dhabi, United Arab Emirates; 4 Sherwood Forest Hospitals NHS Foundation Trust, Nottinghamshire, United Kingdom; 5 Gartnavel General Hospital, Glasgow, United Kingdom; Cardiff University, UNITED KINGDOM

## Abstract

**Background:**

There is a clinical need for therapeutic options to reduce hyperinflation associated with severe emphysema. Endobronchial Coils (coils) are nitinol devices implanted bronchoscopically under fluoroscopic guidance to re-tension the lung. We report the medium term effectiveness and safety of coils in a study of patients with emphysema.

**Methods:**

Forty five subjects with severe airflow obstruction and hyperinflation received bilateral sequential treatment with coils (30 day interval between treatments) as part of a randomised controlled trial with a primary endpoint 90 days after the final treatment (Clinicaltrials.gov NCT01334307). Further assessments were made at 180 and 360 days and in this study the primary outcome was the effect of coil treatment on the St. George’s Respiratory Questionnaire (SGRQ) 360 days following treatment.

**Results:**

At 360 days following treatment, there was an improvement in the SGRQ score of -6.1±14.0 points (p = 0.01) compared to baseline. Improvements in secondary outcomes were seen with increases in forced expiratory volume in the first second of 8.9 ±22.2% (p = 0.002) and 6-minute walking distance of 34.1±52.4m (p = 0.003). The safety profile was acceptable out to 360 days post-treatment.

**Conclusions:**

Statistically and clinically meaningful benefits in quality of life, exercise capacity and pulmonary function in patients treated with coils are sustained twelve months after treatment.

**Trial registration information:**

Clinicaltrials.gov NCT01334307.

## Introduction

Chronic obstructive pulmonary disease (COPD) is now the third leading cause of death globally [1] and has high morbidity and resource utilisation, with a prevalence above 5% in both Europe [2] and the United States.[3] The loss of structural elements, alveolar walls, and pulmonary vasculature in emphysematous lungs leads to a reduction in lung elasticity, over-inflation and premature airway closure during expiration. The result is gas trapping and increased residual volume (RV). During exercise, shorter expiratory times prohibit the expiration of the complete inspired volume from each breath causing progressive worsening of gas trapping, termed dynamic hyperinflation. Thus although bronchodilators can, to a limited extent, improve airflow obstruction, many patients remain breathless despite optimal therapy.

Reducing the volume of the hyperinflated lung to make it a better fit for its thoracic cavity is a sensible approach, particularly so if this is achieved by removing or shrinking the most diseased portions of the lung contributing the least to gas exchange. The most definitive way to achieve this is by surgically resecting the worst affected parts of the lung, known as lung volume reduction surgery (LVRS). This approach is effective in selected patients with upper lobe predominant emphysema and low exercise capacity. However it is associated with significant morbidity and mortality even in this non-high risk subgroup of patients, [4] though more modern surgical techniques and improvements in post-operative care have reduced complications and improved survival.[5, 6] Hence, a drive to develop less invasive methods to achieve the benefits of LVRS has resulted in some promising approaches. Bronchoscopically placed endobronchial valves have had success when targeting the most severely damaged lobe in heterogeneous emphysema, but have limited effect in the presence of collateral ventilation.[7–10] The use of sclerosants [11] or vapour [12] to shrink similar targets in heterogeneous disease has been proposed, with collateral ventilation not an impediment to success;[13, 14] however neither method has undergone evaluation in a large-scale randomised trial. In homogeneous disease, release of trapped gas through extra-anatomical airway stents has been shown to be feasible and safe; however, benefits were short lived due to stent occlusion.[15]

The RePneu Lung Volume Reduction Coils (coils; PneumRx, Mountain View, CA, USA) are shape-memory nitinol devices implanted bronchoscopically using fluoroscopic guidance. They are straightened for deployment and gather up loose parenchyma as they revert to their original double-loop shape within the airway. Multiple coils implanted throughout a lobe achieve mechanical volume reduction through distribution of increased radial tension throughout the airway network, while tethering open small airways to prevent collapse. Effectiveness and a good safety profile have been demonstrated in two small cohorts of patients with heterogeneous emphysema.[16, 17] We conducted a randomised controlled crossover trial of 47 patients randomised in a 1:1 ratio to assess effectiveness and safety, enrolling patients with both heterogeneous and homogeneous disease. The protocol for this trial and CONSORT checklist are available as S1 Protocol and S1 Checklist. Primary endpoint results comparing changes in outcome measures of 23 patients in the coil treatment group and 24 control patients 90 days following final treatment have been reported.[18] In this study we report uncontrolled medium term 180 and 360 day results of all 45 patients treated with coils inclusive of control arm subjects who crossed over to have coil treatment.

## Methods

COPD patients on optimal medical therapy, with severe airflow obstruction, significant hyperinflation and limiting breathlessness, with no contraindications prohibiting bronchoscopy were considered for enrolment. Optimal medical therapy included long acting inhaled anticholinergics and beta agonists in combination with inhaled corticosteroids if tolerated, other pharmacological treatments as appropriate and previous pulmonary rehabilitation with maintenance of regular exercises. The inclusion and exclusion criteria are listed in Table 1. Recruitment took place at three UK sites between the 27^th^ of January 2010 and the 25^th^ of November 2011. The trial was registered with ClinicalTrials.gov, number NCT01334307. After fulfilling all inclusion and exclusion criteria, subjects were randomised in a treatment to control ratio of 1:1 by a computer-generated sequence, as previously described.[18]

**Table 1:** Enrolment criteria.

**Inclusion criteria**
Aged ≥35 years
High resolution computerized tomography (HRCT) scan indicates unilateral or bilateral emphysema
HRCT scan indicates homogeneous or heterogeneous emphysema
A post-bronchodilator forced expiratory volume in the first second (FEV_1_) ≤45% predicted
Total lung capacity >100% predicted
Patient has marked dyspnoea score ≥2 on modified Medical Research Council scale of 0–4
Patient has stopped smoking for a minimum of 8 weeks before enrolment
Patient (or legal guardian if applicable) read, understood, and signed the informed consent form
**Exclusion criteria**
A change in FEV_1_ greater than 20% post-bronchodilator
A single-breath diffusing capacity for carbon monoxide <20% predicted
A history of recurrent clinically significant respiratory infection
Uncontrolled pulmonary hypertension defined by right ventricular pressure >50 mm Hg as evidenced by echocardiogram
An inability to walk >140 metres in 6 minutes
Evidence of other diseases that can compromise survival—e.g., lung cancer or renal failure
Pregnant or lactating
An inability to tolerate bronchoscopy under heavy sedation or anaesthesia
Clinically significant bronchiectasis
Giant bullae greater than a third of lung volume
Previous lung volume reduction surgery, lung transplant, or lobectomy
Participation in other pulmonary drug studies within 30 days of enrolment
Taking greater than 20 mg prednisone (or similar steroid) daily
On clopidogrel or unable to stop treatment for 1 week before the procedure
Other disease that would interfere with completion of study or follow-up assessments, or that would adversely affect outcomes

### Ethics Statement

Approval was obtained from the London-Chelsea National Research Ethics Service committee (reference 09/H0708/51) and all patients provided written informed consent.

### Treatment and assessment visits

**Fig 1:**
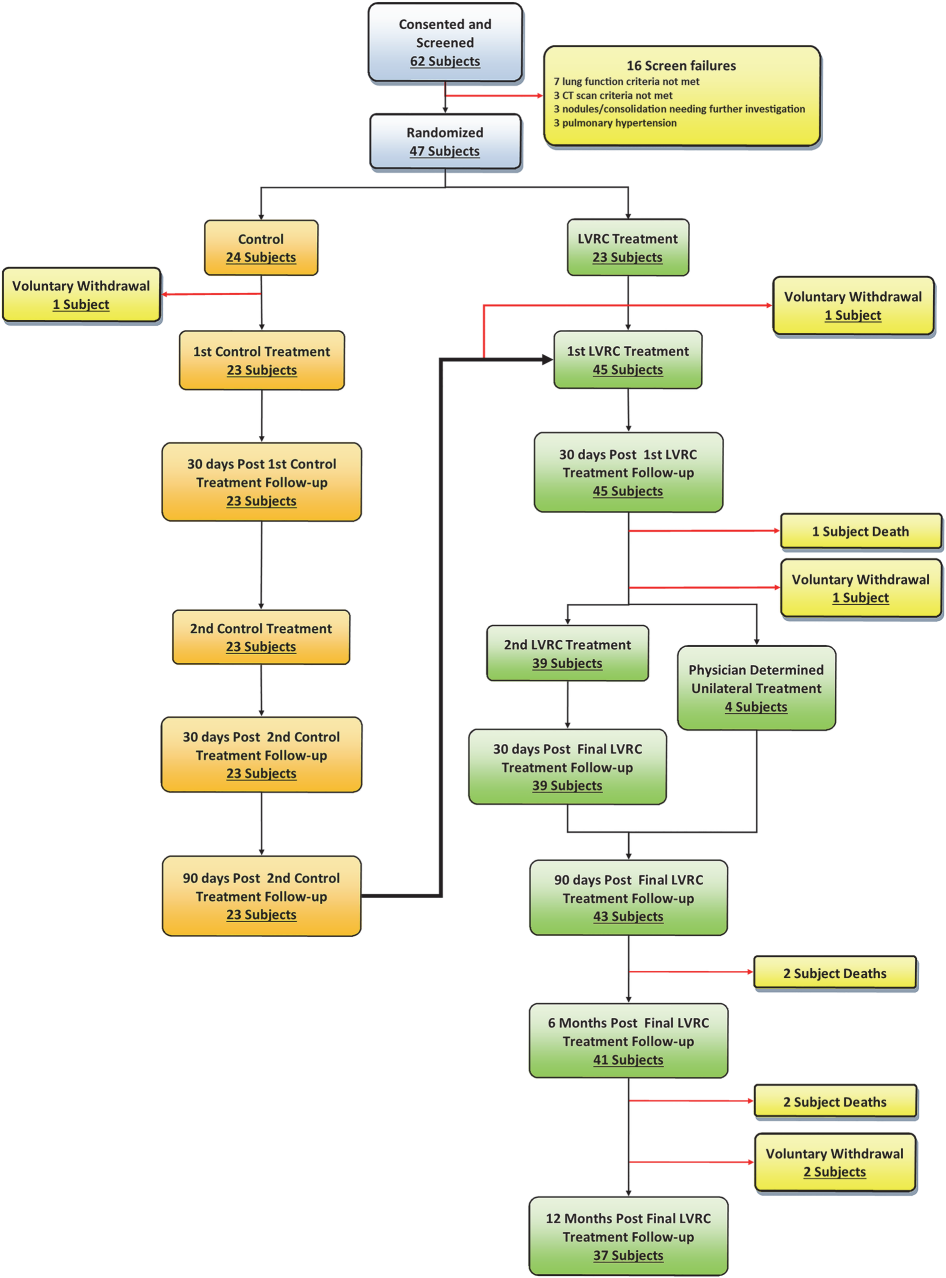
Flow diagram for the study. The primary endpoint in this study is the change in St. George’s Respiratory Questionnaire (SGRQ) [19] at 360 days post final treatment. A change of four points is widely accepted to be the minimally clinically important difference (MCID).[20] Pulmonary function tests including whole body plethysmography were performed as per international guidelines using the European Community of Coal and Steel Workers’ cohort normal values.[21] The 6 minute walk distance (6MWD) was performed according to ATS guidelines.[22] These assessments were performed at each non-treatment visit.

The consort diagram in Fig 1 details the study schedule and subject numbers at each time point. In summary, subjects entered the treatment arm either immediately after enrolment or after 157 ± 34 days in the control phase. Patients had the worst affected lobe in the first lung treated and were reviewed 30 days later. If there were no contraindications, the worst affected lobe in the contralateral lung was treated and the patient reviewed 30, 90, 180 and 360 days after the final treatment.

Dedicated high-resolution computerized tomography (HRCT) scan densitometry analysis software (Pulmo-CMS Version 2.1.5; Medis Specials, Leiden, Netherlands) was used to assess HRCT scans (SOMATOM Definition AS and Sensation 64; Siemens Medical, Erlangen, Germany), providing a density map of the lungs. This allowed assessment of severity of bullous destruction for trial exclusion, and assessment of heterogeneity for treatment planning.[18] A lobar 0 to 5 qualitative scoring system for emphysema severity was used. In brief: 0-score: no parenchymal damage; 1-score: mild centrilobular damage with 1-3mm bullae; 2-score: centrilobular damage with max 5-10mm bullae; 3-score: blebs and/or small bullae max 10-20mm; 4-score, significant pan lobular damage including bullae 20-50mm in diameter; 5-score: severe bullous disease and lobes presenting with little remaining lung structure. The major lobes in each lung were compared, and if the score differed by 0–1 point, the lung was considered homogeneous; if the score differed by ≥2 points, the lung was considered heterogeneous. If one or both lungs were heterogeneous, the patient was considered heterogeneous. HRCT scans were performed at screening, and at 90 and 180 days following final treatment.

### Procedures

Bronchoscopy was performed using moderate sedation unless the subjects requested general anaesthesia. A guide wire was carefully passed into the target subsegmental airway and advanced under fluoroscopic guidance, avoiding sharp changes of direction, to a distance still safely away from the pleural edge (>35mm). The delivery catheter was then advanced over the guide wire until the two were aligned. Fluoroscopically visible markers on the guide wire allowed coil sizing. The appropriate sized coil was prepared for deployment by extracting it directly into a straight deployment cartridge using specialised forceps. The cartridge was coupled to the delivery catheter and the coil advanced through the bronchoscope and into the target subsegment. Under fluoroscopic guidance, the coils were advanced until the distal tip reached the distal end of the delivery catheter, and the sheath was then pulled back, releasing the coil, which regained its pre-determined shape. Up to 14 coils were distributed evenly throughout the target lobe to achieve maximal regional tension in the treated area. A post-procedure chest radiograph was performed to rule out pneumothorax, and each patient was observed overnight.

### Analysis

Data analyses of primary and secondary endpoints of the randomised controlled trial were pre-specified in a formal Statistical Analysis Plan (SAP) and previously reported.[18] Formal analyses of the data provided herein (medium term follow-up including data from cross-over treatments) were not pre-specified, thus, descriptive statistics were used to summarise the data. The primary outcome measure here is the change in SGRQ at 360 days post final treatment as compared to baseline. Statistical significance was assessed by a student’s t-test, two-sample t-test, or Fisher’s exact test as appropriate. Comparisons were made between baseline and 90, 180 and 360 days post final treatment in order to assess persistence of effect over the medium term. Least squares regression was used to compare changes in clinical outcomes with the number of implanted coils in univariate analysis, then multivariate least squares regression fitting clinical outcomes to number of coils.

For control group patients who crossed-over to the treatment arm, the 90 day post final control treatment visit outcome values were used as their baseline values as they joined the coil treatment arm.

Before acceptance of the clinical data, a monitoring team independent of the Sponsor performed 100% source verification of the recorded clinical data. Monitored data were double entered and stored by the sponsor. All data analyses were conducted using SAS Version 9.3.

## Results

Initially 24 subjects were randomised to the control arm and 23 to the treatment arm. Following crossover, the number of patients treated with coils was 45 (one control group subject withdrew before her first control treatment visit, and another after completing control follow-up but before his first coil treatment as he did not feel that he could continue to regularly travel the large distances from his home to the research site (Fig 1).

Baseline characteristics and demographics are detailed in Table 2. All but six procedures (in three patients, 78/84) were performed using local anaesthetic and moderate sedation (topical lidocaine, and intravenous midazolam and fentanyl). Three patients requested general anaesthesia. Procedure time was 41.6±15.8 minutes. A median (range) of 9 (5 to 14) coils were implanted per procedure, per treated lung. The median hospital stay was 1 day. Discharge the following day as per study protocol occurred in 76 of the 84 treatments (90.5%), with discharge delayed on 8 occasions (6 delays due to pneumothorax requiring chest drain; and on 2 occasions due to exacerbations of symptoms). The 90, 180 and 360 day post final treatment visits took place 134 ±18, 225 ± 20, and 409 ± 24 days after the first treatment, respectively.

**Table 2:** Baseline characteristics.

**Baseline Measure**	**Coil Treatment Group (N = 45)**
**Gender- % (n/N)**	
Male	62.2% (28/45)
Female	37.8% (17/45)
**Age (years)**	63.8 ±7.9
**Body Mass Index (kg/m** ^2^ **)**	24.4 ±4.8
**Emphysema Distribution- % (n/N)**	
Heterogeneous	33.3% (15/45)
Homogeneous	66.7% (30/45)
**Forced Expiratory (L)- 1 Second**	0.76 ±0.20
**Forced Expiratory—1 Second- % Predicted**	28.3 ±8.0
**Residual Volume—% Predicted**	224.9 ±49.9
**Total Lung Capacity—% Predicted**	135.1 ±15.4
**Residual Volume / Total Lung Capacity—%**	62.1 ±6.9
**Forced Vital Capacity (L)**	2.75 ±0.59
**Forced Vital Capacity- % Predicted**	82.0 ±16.4
**Carbon monoxide diffusing capacity—% Predicted**	35.3 ±10.6
**Six Minute Walk Distance (m)**	310.1 ±82.9
**St. George's Respiratory Questionnaire- Total Score (points)**	58.9 ±13.2
**Supplemental Oxygen- % (n/N)**	28.9% (13/45)
**mMRC Dyspnea Scale**	
0	0.0% (0/45)
1	2.2% (1/45) ^¥^
2	51.1% (23/45)
3	40.0% (18/45)
4	6.7% (3/45)

^¥^ One patient with an mMRC score of 2 on study enrolment had an mMRC of 1 after crossing over from the control phase.

Evaluable data was available for 43 patients 90 days following treatment (one patient withdrew from the study after their first treatment and one patient died before their 90 day follow-up visit), 41 patients 180 days following the final treatment (two patients died before their 180 day follow-up visit) and for 35 patients 360 days following final treatment (two patients died, two patients were unwell with infective exacerbations and their follow-up tests took place outside of the acceptable window for inclusion in the analysis, one patient withdrew from the study, and one patient was lost to follow-up before their 360 day follow-up visits).

**Fig 2:**
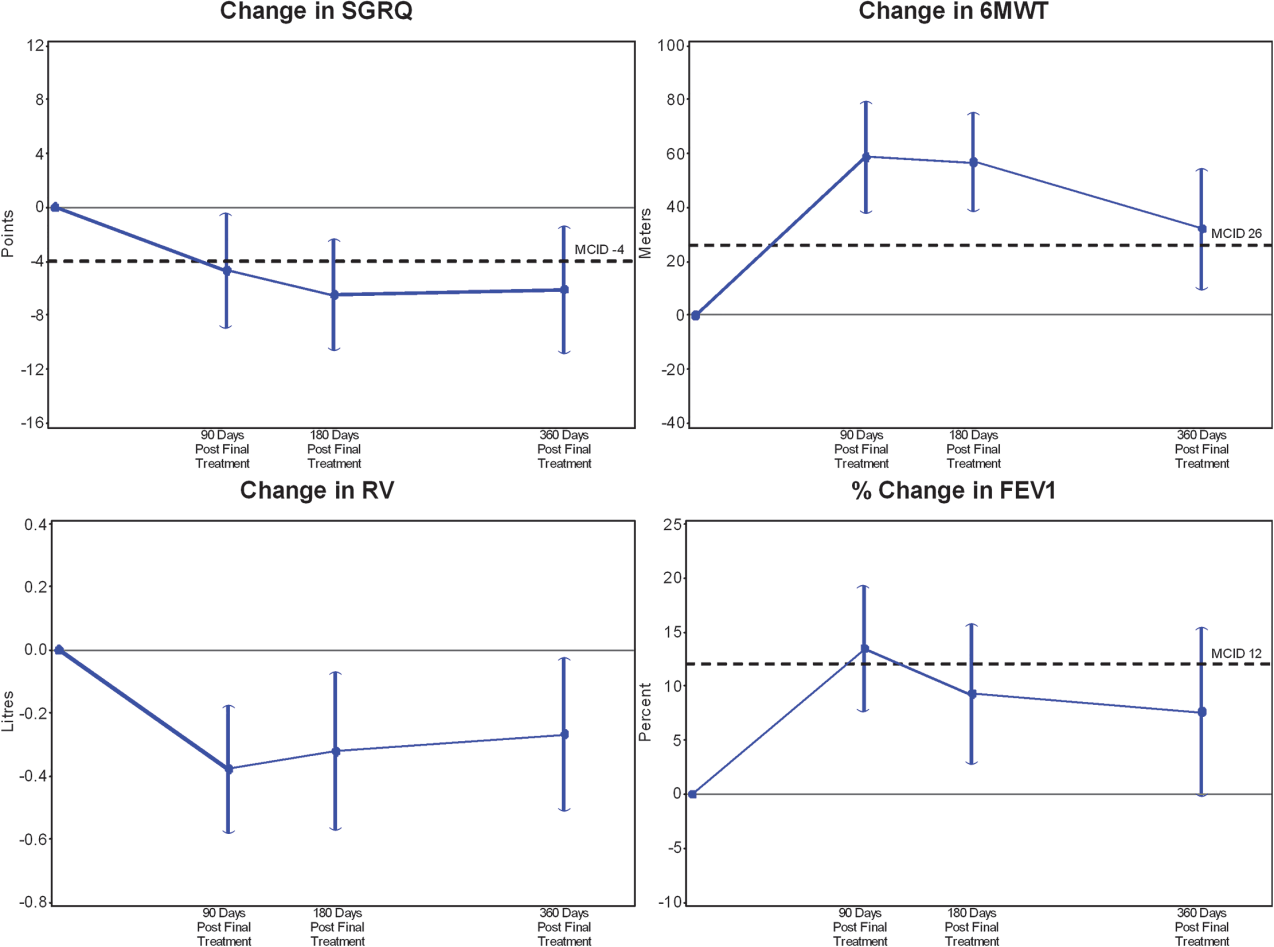
Efficacy outcomes in the coil treated patients 90, 180 and 360 days post final treatment.

Fig 2 illustrates the efficacy outcomes in the coil treated patients 90, 180 and 360 days post final treatment. The treatment group had a -6.1±14.0 (p = 0.01) point improvement in the SGRQ 360 days after final treatment compared to baseline, with clinically and statistically significant benefits at all time points (Table 3). Exercise capacity, an important patient centred outcome, also improved significantly at all time points compared to baseline (Table 3). Improvements in lung function were seen with a 90±120 ml increase in the FEV_1_ 90 days following the final treatment as compared to baseline (p = <0.0001), representing a 13.8±18.1% increase (p<0.0001). Benefits were maintained with 10.0±21.0% (p = 0.005) and 8.9±22.1% (p = 0.024) increases 180 and 360 days after treatment, respectively, as compared to baseline. RV and RV/total lung capacity (TLC) ratio, but not TLC, were significantly reduced at all time points as compared to baseline (Table 3).

**Table 3:** Efficacy outcomes in coil treated patients 90, 180 and 360 days after final treatment as compared to baseline.

**Efficacy outcomes**	**90 days post final treatment (N = 43)**		**180 days post final treatment (N = 41)**		**360 days post final treatment (N = 35)**	
	**Difference from Baseline**	**Percent Change From Baseline**	**Difference from Baseline**	**Percent Change From Baseline**	**Difference from Baseline**	**Percent Change From Baseline**
**St. George's Respiratory Questionnaire- (**Points)	-4.7 ±13.4 (-6.6)		-7.3 ±12.2 (-5.3)		-6.1 ±14.0 (-5.8)	
p-value	0.02		0.0005		0.01	
**Forced Expiratory 1 Second** (L)	0.09 ±0.12 (0.09)	13.8 ±18.1 (11.9)	0.07 ±0.15 (0.09)	10.0 ±21.1 (11.0)	0.06 ±0.17 (0.02)	8.9 ±22.2 (3.9)
p-value	< 0.0001	< 0.0001	0.008	0.005	0.04	0.02
**Forced Vital Capacity** (L)	0.23 ±0.37 (0.20)	9.5 ±14.1 (9.2)	0.21 ±0.47 (0.20)	9.6 ±18.4 (7.4)	0.19 ±0.44 (0.20)	8.4 ±16.3 (7.0)
p-value	0.0002	< 0.0001	0.008	0.002	0.02	0.004
**Residual Volume** (L)	-0.38 ±0.61 (-0.47)	-7.1 ±10.5 (-9.4)	-0.34 ±0.79 (-0.30)	-5.8 ±13.6 (-5.4)	-0.32 ±0.77 (-0.22)	-5.4 ±13.7 (-5.2)
p-value	0.0003	< 0.0001	0.009	0.01	0.02	0.03
**Total Lung Capacity** (L)	-0.16 ±0.41 (-0.10)	-1.8 ±5.2 (-1.2)	-0.16 ±0.52 (-0.10)	-1.7 ±6.2 (-1.6)	-0.13 ±0.48 (-0.10)	-1.4 ±6.1 (-1.2)
p-value	0.02	0.03	0.06	0.09	0.12	0.19
**Residual Volume/Total Lung Capacity (%)**	-3.59 ±4.82 (-3.3)	-5.5 ±7.6 (-5.6)	-3.06 ±6.05 (-2.2)	-4.6 ±9.3 (-2.0)	-2.93 ±6.35 (-3.0)	-4.3 ±10.0 (-6.9)
p-value	< 0.0001	< 0.0001	0.003	0.004	0.01	0.01
**Six Minute Walk Distance (**m)	56.0 ±65.1 (52.5)	20.3 ±23.8 (20.8)	54.6 ±54.2 (49.5)	20.2 ±20.8 (19.1)	34.1 ±52.4 (35.0)	14.0 ±20.8 (14.1)
p-value	< 0.0001	< 0.0001	< 0.0001	< 0.0001	0.0005	0.0003

The 90, 180 and 360 day post final treatment visits took place 96 ± 10 days, 186 ± 10 days and 367 ± 17 days after the final treatment, respectively.Data presented as mean ± SD (median).

Patients with both heterogeneous (32.1% of treated lungs, 27/84) and homogeneous (67.9% of treated lungs, 57/84) emphysema were recruited. No significant differences were seen in changes in efficacy outcomes between patients with bilateral heterogeneous disease (n = 14) and those with homogeneous disease (n = 21) 360 days following treatment compared to baseline: ΔSGRQ -5.9 ± 13.1 vs. -6.2±15.2, p = 0.961; %ΔFEV_1_ 6.9 ± 18.8 vs. 10.1 ± 24.5, p = 0.831; ΔRV -0.45 ± 0.84 vs. -0.23 ± 0.73, p = 0.652; and Δ6MWD 39.8 ± 51.2 vs. 30.3 ± 54.2, p = 0.639), respectively.

### Adverse Events

There were eight serious adverse events (SAE) in eight patients related to respiratory tract infections or COPD exacerbations in the 30 days following the 84 treatments (9.5% of procedures), including one pneumonia which resolved with outpatient treatment with oral antibiotics (consolidation in the vicinity of coils seen on chest radiograph) (Table 4). A further 12 respiratory SAEs occurred in 11 patients in the time period of 31 days post treatment to 360 days post treatment (none of these were pneumonia). A total of eight pneumothoraces were reported. Six occurred within four hours of the procedure (only one during the procedure confirmed with fluoroscopic screening), and all six were managed with 12 French gauge intercostal tube drainage. All resolved without further intervention and discharge was delayed by 2.8 ±1.0 days. One patient had a recurrence of his pneumothorax 78 days following the original event, and this again resolved following intercostal drainage for 10 days. This patient has not had further recurrence after more than 720 days of follow-up. Another patient presented with a pneumothorax 31 days following his procedure and this resolved with large bore intercostal chest tube drainage with suction applied for 19 days.

**Table 4:** Investigator reported respiratory serious adverse events.

**Serious Adverse Event**	**Coil Group**			
**Treatment Recovery Period (0–30 days post procedure)**	**Number of Events**		**Number of Subjects**	**Percent of Procedures**
Bronchospasm	0		0	0.0% (0/ 84)
COPD Exacerbation	4		4	4.8% (4/ 84)
Chest pain	1		1	1.2% (1/ 84)
Device Removal	0		0	0.0% (0/ 84)
Haemoptysis	0		0	0.0% (0/ 84)
Lower respiratory tract infection	3		3	3.6% (3/ 84)
Pneumonia	1		1	1.2% (1/ 84)
Pneumothorax	5		5	6.0% (5/ 84)
**Subsequent Post-Treatment Period (>30 Days)**				
Bronchospasm	0		0	0.0% (0/ 84)
COPD Exacerbation	5		4	6.0% (5/ 84)
Chest pain	1		1	1.2% (1/ 84)
Device Removal	0		0	0.0% (0/ 84)
Haemoptysis	0		0	0.0% (0/ 84)
Lower respiratory tract infection	1		1	1.2% (1/ 84)
Pneumonia	1		1	1.2% (1/ 84)
Pneumothorax	3		2	3.6% (3/ 84)

Five deaths occurred within the period extending to 360 days after treatment. One patient developed a severe infective exacerbation of their airways disease secondary to influenza B, and died following a haemorrhagic stroke whilst ventilated on the intensive care unit. One patient developed a severe infective exacerbation of his COPD approximately 10 weeks after his second treatment and died from respiratory failure refractive to ventilatory therapy via endotracheal tube at the end of a two-week hospital admission. One patient died secondary to a severe infective exacerbation of COPD seven months after his final treatment. Although one cannot be certain that the presence of coils within the airways did not influence onset of or treatment response to these exacerbations, the frequency and time courses of these three adverse events is not unexpected for such patients with end stage COPD. One patient died from severe urinary sepsis 335 days after coil treatment and one patient died from oesophageal cancer 309 days after the second coil treatment. All five deaths occurred in the group originally randomised to the control arm.

## Discussion

Treatment with coils resulted in significant improvements in quality of life, exercise capacity, and lung function with benefits maintained for at least one year compared to baseline. The improvement in the primary endpoint, change in SGRQ, was greater than the widely accepted minimally clinically important difference (MCID) of 4 points.[20] Although SGRQ is a self-reported measure and patients were not blinded to the fact that they had received coil treatment, this change was accompanied by clinically important improvements in lung function and exercise capacity, suggesting a true rather than placebo effect. The persisting improvements out to 409 ± 24 days after the first treatment further support a true benefit.

Improvements in the 6MWD also exceeded the MCID of 26 metres that has been established in the context of lung volume reduction procedures,[23] though there was some loss of benefit from a peak of Δ+56.0±65.1m 90 days post final treatment to Δ+34.1±52.4m 360 days post final treatment. Patients experienced improvements in lung function consistent with reduction in gas trapping and airways obstruction, but not total lung capacity. This is in contrast with LVRS and lung volume reduction with endobronchial valves, where there is loss of lung parenchyma and reduction in TLC. Thus the mechanisms of action of the coils probably relate to prevention of dynamic airway collapse and reduction in gas trapping rather than leading to volume reduction *per se*. This effect has a greater impact on dynamic hyperinflation which is reflected in the improvements in exercise capacity.[24]

As with the 6MWD, there was a small trend back towards baseline in the reduction in RV and increase in FEV_1_, but values remained statistically and clinically significantly better than baseline 360 days post final treatment. Emphysema is a progressive disease and a gradual deterioration in lung function parameters over time is expected. Controlled data at 12 months post treatment is required to comment definitively on durability of benefit from coils. The Endobronchial Valves for Emphysema Palliation Trial (VENT) [7] reported data on a randomised, controlled population of severe emphysema patients with similar baseline characteristics, undergoing optimal medical care, providing an estimate of functional decline expected in this patient group. In these control patients, median change from baseline to 6 months in 6MWD was -10.7 (-29.6 to 8.1) m, in SGRQ +0.6 (-1.8 to 3.0) points, and in absolute percent change in FEV_1_ of -2.4 (-5.1 to 0.4). [7] These small but significant reductions are expected given the compromised status of patients at randomisation and the progressive nature of the disease. It is not possible to predict the rate of deterioration towards baseline in the efficacy outcomes beyond 12 months (completion of trial follow-up), and there is a need for longer-term controlled studies to establish the true magnitude of benefit of coil treatment in this patient group.

The overall SAE profile was acceptable given the benefits accrued, and compares favourably with the SAE profile and morbidity associated with lung volume reduction surgery. Although seven patients experienced SAE pneumothoraces (8.3% of procedures, 15.5% of patients) within the 360 day follow up period, none required surgical intervention and the majority resolved within 4 days with simple intercostal tube drainage. The pneumothoraces were evenly spread out amongst early, middle and late recruits to the study, making a “learning effect” an unlikely cause. Following treatment of the pneumothorax, these seven patients achieved a mean reduction in the SGRQ of -8.8 points, an increase in FEV1 of 0.09L, a reduction in the RV of -0.62L and increase in the 6MWD of 42.9m, 360 days following coil treatment. Hence the occurrence of a pneumothorax does not preclude benefit.

LVRS and other bronchoscopic lung volume reduction treatments are most effective when disease is heterogeneously distributed with a clear anatomical target with worse disease. In this study of endobronchial coils, we recruited patients with both heterogeneous and homogeneous emphysema with both groups benefiting equally from coil treatment. The percentage split between heterogeneous and homogeneous disease in this cohort likely approximates the rates of heterogeneous vs. homogeneous disease in severe emphysema populations in the United Kingdom, and at present there is no alternative effective interventional treatment option for patients with homogeneous emphysema. Coil treatment appears particularly promising in light of its potential to treat this significant group of patients.

The median number of coils implanted per bilaterally treated patient was 18 (range 13–27) coils in this study. There was no relationship between the number of coils implanted per patient and change in efficacy outcomes 360 days following treatment on univariate or multivariate analysis: Number of coils vs. ΔSGRQ r^2^ 0.083, p = 0.10; vs. ΔRV r^2^<0.0001, p = 0.96; vs. %ΔFEV_1_ r^2^ = 0.013, p = 0.53; and vs. Δ6MWD r^2^ = 0.02, p = 0.45).

One limitation of this trial is the cross-over design and potential for randomisation bias. There were no significant differences in changes in outcome measures between the original treatment group (n = 21) and the crossover group (n = 16) 360 days following treatment compared to baseline: ΔSGRQ -7.13 ± 11.91 vs. -4.75 ± 16.50, p = 0.64; %ΔFEV_1_ 7.6 ±22.9 vs. 10.3 ± 23.5, p = 0.85; ΔRV -0.28 ± 0.82 vs. -0.36 ± 0.72, p = 0.87; and Δ6MWD 35.3 ± 55.5 vs. 32.6 ± 50.0, p = 0.67.

Evaluable data at 360 days were available for 35 of the 45 treated patients. Hence we examined whether there were differences in baseline values and outcomes when comparing the group that had 360 day follow-up assessments and the 10 patients who did not (5 deaths and 5 drop outs). There were no differences in baseline values and the primary efficacy outcome change in SGRQ was no different between these two groups at 90 days (ΔSGRQ -4.3 ± 14.2 vs. -7.8 ± 18.7, p = 0.99) and 180 days (ΔSGRQ -7.0 ± 11.8 vs. -9.7 ± 16.4, p = 0.68), making it unlikely that the drop outs positively biased the group 360 day outcome data.

## Conclusion

Treatment with coils results in improvements in quality of life, exercise tolerance and lung function, which are sustained up to 360 days post treatment. Overall, the safety profile is acceptable particularly in view of the magnitude of benefit and in comparison with surgical and other bronchoscopic lung volume reduction techniques. The coil method may be particularly promising for patients with relatively homogeneous emphysema, who currently have no other treatment options, and appears to be effective across a diverse population of emphysema patients.

## Supporting Information

S1 Checklist CONSORT Checklist. (DOC)

S1 Protocol Endobronchial coils for the treatment of severe emphysema with hyperinflation (RESET) trial study protocol. (PDF)
