# Supplementary material for: Endobronchial Coils for Severe Emphysema Are Effective Up to 12 Months following Treatment: Medium Term and Cross-Over Results from a Randomised Controlled Trial
Source: PLoS One. 2015 Apr 8;10(4):e0122656. doi: 10.1371/journal.pone.0122656 (PMC4390222; doi:10.1371/journal.pone.0122656)
Supplement: S1 Protocol — (PDF) [file pone.0122656.s002.pdf]

PneumRx

CLINICAL TRIAL PROTOCOL COVER SHEET

Device: PneumRx, Inc. Lung Volume Reduction Coil

Study Number & Rev.: CLN0008-01.p.D

Study Title: Randomized Comparison of the PneumRx, Inc. Lung Volume Reduction Coil to Standard of Care for the Treatment of Emphysema

Study Design: Multicenter, randomized, controlled study of safety and performance for PneumRx, Inc. Lung Volume Reduction Coil

Sponsor Name: PneumRx, Inc.

Sponsor Address: 530 Logue Avenue
Mountain View, California 94043
USA

Authorized Representative: MPS Medical Product Service GmbH
Borngasse 20
35619 Braunfels
Germany
Tel 49 (0) 6442 962073
Fax 49 (0) 6442 9622074

Study Coordination and Data Analysis: PneumRx, Inc.

Projected Initiation Date: December, 2009

Projected Completion Date: December, 2011

STATEMENT OF CONFIDENTIALITY

The information contained herein is confidential information that is the sole and exclusive property of PneumRx, Inc. and may not be divulged to any person (except as required by law) without the prior written consent of PneumRx, Inc.

Revision History			
**Revision**	**CCO**	**Effective Date**	**Originator**
A	0033	October 21, 2008	Jeff Rondinone
B	0034	December 1, 2008	Jeff Rondinone
C	0035	December 1, 2008	Jeff Rondinone
D	0048	October 13, 2009	Jeff Rondinone

____

Joseph Rondinone, PhD
Vice President, Clinical and Regulatory Affairs
PneumRx, Inc

____

Erin McGurk
President, CEO
PneumRx, Inc

STUDY ACKNOWLEDGMENT

Investigator's Statement:

I have read and understand the foregoing Protocol No. CLN0008-01.p.C and any corresponding amendments entitled ”Randomized Comparison of the PneumRx, Inc. Lung Volume Reduction Coil to Standard of Care for the Treatment of Emphysema” and agree to conduct the study as outlined herein.

____
Investigator's Name (please print)

____
Investigator’s Title

____
Investigator's Signature

____
Date

Sponsor Signature:

For: PneumRx, Inc.
530 Logue Avenue
Mountain View, CA 94043
USA

____
Name (please print)

____
Signature

____
Position/Title

____
Date

Table of Contents

1 Introduction 7
1.1 Background 7
1.2 Clinical Need 7
1.3 Description of the PneumRx, Inc. Lung Volume Reduction Coil 8
2 Report of Prior Investigations 11
3 Study Objectives 18
3.1 Primary efficacy endpoint: 18
3.2 Secondary efficacy endpoints: 18
3.3 Safety Objectives: 18
3.4 Safety Analysis: 18
4 Study Design 19
4.1 Design Overview 19
4.2 Number of Subjects 19
4.3 Treatment Group 19
4.4 Control Group 19
4.5 Population 19
4.6 Demographic and Baseline Characteristics 19
4.7 Safety Evaluation 20
4.8 Brief Description of Study 20
5 Study Subject Recruitment 24
5.1 Inclusion Criteria 24
5.2 Exclusion Criteria 24
6 Study Plan 25
6.1 Test Device 25
6.2 Detailed Study Description 25
6.2.1 Informed Consent 25
6.2.2 Study Identification Number 25
6.2.3 Screening Period 25
6.2.4 Screening Evaluations 25
6.2.5 Randomization 26
6.2.6 "Treatment #1" **(Control Patients Only)** 26
6.2.7 1 Week Post "Treatment #1" Follow Up Phone Call 26
6.2.8 1 Month Post "Treatment #1" Follow Up Evaluation 26
6.2.9 Post 1 Month Follow Up 27
6.2.10 "Treatment #2" **(Control Patients Only)** 27
6.2.11 1 Week Post "Treatment #2" Follow Up Phone Call 27

6.2.12 1 Month Post “Treatment #2” Follow Up Evaluation 27
6.2.13 3 Month Post-“Treatment#2” Follow Up Evaluation 27
6.2.14 Bronchoscopy #1 / LVRC Placement 28
6.2.15 Post Bronchoscopy Monitoring and Evaluations 28
6.2.16 1 Day Hospital Stay 28
6.2.17 1 Week Post-Bronchoscopy #1 Follow Up Phone Call 28
6.2.18 1 Month Post-Bronchoscopy #1 Follow Up Evaluation 29
6.2.19 Post 1 Month Follow Up Second Treatment Option 29
6.2.20 Bronchoscopy #2 / LVRC Placement 29
6.2.21 Post Bronchoscopy Monitoring and Evaluations 30
6.2.22 1 Day Hospital Stay 30
6.2.23 1 Week Post Bronchoscopy #2 Follow Up Phone Call 30
6.2.24 1 Month post-bronchoscopy #2 Follow Up Evaluation 30
6.2.25 3 Month Post-Bronchoscopy #2 Follow Up Evaluation 30
6.2.26 Study Exit 31

7 Adverse Events 32

8 Risks and Benefits 34

8.1 Potential Risks to the Patient 34

8.2 Potential Benefits to the Patient 36

9 Study Monitoring 37

10 Responsibilities of the Sponsor 38

11 Responsibilities of the Principal Investigator 39

12 Good Clinical Practice & Regulatory Requirements 41

13 References 42

List of Abbreviations and Definitions of Terms

AE	Adverse Event
BD	Bronchodilator
CRF	Case Report Form
CXR	Chest X-ray
DLCO	Diffusion Capacity of the Lung for Carbon Monoxide
EC	Ethics Committee
ECG	Electrocardiogram
FEV_1	Forced Expiratory Volume (in one second)
GCP	Good Clinical Practice
GLP	Good Laboratory Practices
HRCT	High Resolution Computed Tomography (CT Scan)
IC	Informed Consent
IFU	Instructions for Use
IRB	Institutional Review Board
LVRC	Lung Volume Reduction Coil (formerly called the Lung Volume Reduction Device (LVRD)
LVRD	Lung Volume Reduction Device
LVRS	Lung Volume Reduction Surgery
mMRC	Modified Medical Research Council
O_2	Oxygen
QOL	Quality of Life
RV	Residual Volume
SAE	Serious Adverse Event
SOP	Standard Operating Procedure
SpO_2	Oxygen Saturation by pulse oximetry
SGRQ	St. George's Respiratory Questionnaire
UADE	Unanticipated Adverse Device Effect

1 Introduction

1.1 Background

The objective of this study is to demonstrate the safety and performance of the PneumRx, Inc. Lung Volume Reduction Coil (LVRC) in a population of patients with emphysema. The PneumRx, Inc. LVRC is used as a less invasive alternative to lung volume reduction surgery.

Physician-investigators who wish to participate in this study understand that the study will be conducted under all applicable regulatory requirements for the country where the study is being conducted. All participating investigators and co-Investigators will be asked to sign a sponsor generated Investigator's Agreement (Appendix A), as well as any required country specific Investigator's Agreement.

This study is being conducted according to Good Clinical Practices (GCP), in compliance with the principles enunciated in the Declaration of Helsinki (World Medical Association Declaration of Helsinki, 2000), applicable local regulations, and per PneumRx, Inc. Clinical Department Standard Operating Procedures (SOPs).

1.2 Clinical Need

Emphysema is a serious disease that affects an estimated 60 million people around the world. It causes debilitating illness and can result in death. Emphysema is a chronic respiratory disease that causes over-inflation of the air sacs (alveoli) in the lungs, causing a decrease in lung function, and often, breathlessness (dyspnea). Normally functioning lungs are elastic, efficiently expanding and recoiling to drive air freely through the bronchus to the alveoli as the patient inhales and exhales. In the emphysematous lung, tissue is damaged and loses its elasticity. This also causes over inflation of the lung, or 'hyperinflation'. When the lungs are hyperinflated, they can no longer expand and contract properly and the chest begins to expand. Small passageways leading to the alveoli collapse, trapping air within the alveoli. Additionally, emphysema causes alveolar walls to be destroyed, creating enlarged air pockets that communicate, resulting in collateral ventilation. The alveolar surface area decreases, reducing the amount of gas exchange that can take place. As a result, the emphysema patient eventually becomes hypoxemic, resulting in muscle weakness and fatigue. The crippling effects of end-stage emphysema include, coughing, severe dyspnea, severe limitation of activities, illnesses, lung infections and can result in death.

There are several medical treatments available for emphysema. Emphysema can be treated with bronchodilators, theophylline and supplemental oxygen. Emphysema patients are prone to respiratory infections and are often prescribed antibiotics. Some patients may have pulmonary rehabilitation exercises and training. There are also two surgical procedures available: lung transplantation surgery and lung volume reduction surgery (LVRS). Lung transplant surgery is an unrealistic option for many emphysema patients because there is a shortage of available donor lungs. Lung Volume Reduction Surgery is major surgery that carries the risk of high morbidity and mortality. Recently less invasive bronchoscopic approaches have been developed and several approaches are being actively investigated in human clinical trials in Europe and the US.

The PneumRx, Inc. Lung Volume Reduction Coil (LVRC) is designed as an alternative to lung volume reduction surgery, potentially achieving the desired reduction in lung volume, while limiting the risks associated with major surgery, such as illness or death. This device is deployed through a bronchoscope and requires no incision.

1.3 Description of the PneumRx, Inc. Lung Volume Reduction Coil

The PneumRx, Inc. LVRC (formerly called the PneumRx LVRD) is an implantable device, delivered through a bronchoscope, designed specifically for patients suffering from emphysema. The LVRC is a two part system that consists of 1) sterile permanent implants, and 2) a sterile, disposable, single-use (single-patient) delivery system consisting of a cartridge, a catheter, a guide wire, and forceps.

The implant is a coil-like device composed of Nitinol, a super-elastic memory shape alloy. The self-actuating implant is delivered straight into airways and recovers to a non-straight, pre-determined shape upon deployment. The intended physiological benefit of the coil implant will be similar to the effects of LVRS, by bending the airways and compressing diseased lung parenchyma to reduce lung volume. The implant is intended to tension the surrounding tissue, which increases elastic recoil and redirects air to healthier portions of the lung for more effective ventilation. This therapy targets local diseased regions of the lung; therefore, one or more implants may be necessary to achieve adequate effect. The LVRC implants will effectively reduce lung volume, even when the affected parenchyma contains collateral ventilation.

The implant derives its strength from the Nitinol wire. The implants are available in seven lengths to accommodate anatomical variations – the lengths are 70 mm, 85 mm, 100 mm, 125 mm, 150 mm, 175mm and 200 mm. Ten millimeters (10 mm) of the proximal end of the implant is floppy, to minimize trauma on the airway wall. The distal and proximal ends of the implant are designed to reside in airways with an inner diameter (ID) of two millimeters (~2 mm) to six millimeters (~6 mm), respectively.

The purpose of the delivery system is to safely deliver the implants. The guide wire serves as a specialized guide for the catheter by identifying ideal airways to treat and supporting the catheter to help guide it to a delivery site. The guide wire also facilitates the selection of the appropriate implant length. The catheter functions as a conduit to deliver the implant from outside the patient to the targeted treatment area. It also can also be used to reposition or remove the implant. The cartridge straightens the implant, couples to the catheter, and aids in the process of loading the implant into the catheter. The forceps couples to the proximal end of the implant and delivers it through the catheter, enabling the clinician to control the placement and release of the device.

The implant can be removed by reversing the deployment procedure: re-capturing the proximal end of the implant with the forceps and then advancing the catheter distally over the implant while maintaining the relative position of the implant to the bronchoscope.

The procedure is designed to be performed using a therapeutic bronchoscope, with a 2.8 mm working channel, and fluoroscopy for visualization beyond the bronchoscope.

Each implant is individually pouched in its own protective packaging shell and five implants of the same size are packaged in a box. The cartridge, catheter, guide wire, and forceps are pouched together and packaged in a box. The LVRC is sterilized by Ethylene Oxide (EO) or E-Beam.

Figure 1. Shapes and Sized of available LVRD Implants.

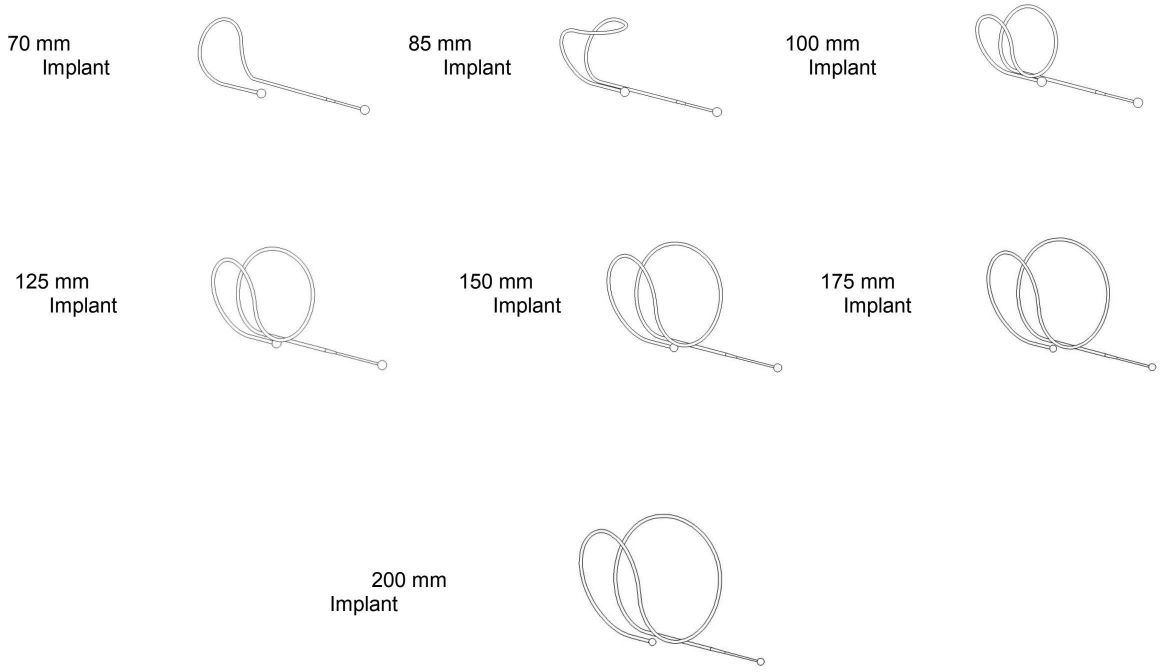

Figure 2. Components of the Delivery System

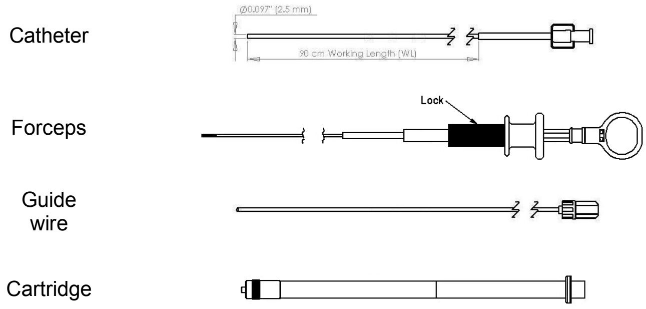

Figure 3. Diagram of the Lung Volume Reduction Procedure Using LVRC Implants

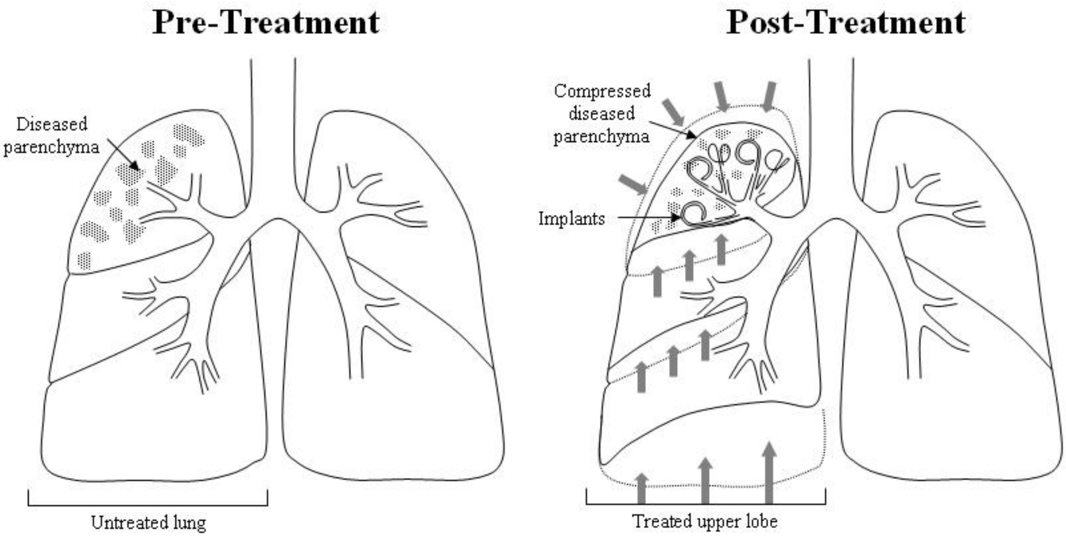

2 Report of Prior Investigations

The PneumRx, Inc. LVRC has undergone careful and comprehensive pre-clinical studies. The safety and effectiveness of the PneumRx, Inc. LVRC is being evaluated in human patients under this protocol.

Summaries of preclinical studies and the first clinical study are presented below.

- **LVRD Biocompatibility Study (VVQ-0069)**

Title: *Testing for Implant and Externally Communicating Devices*

Initiated: April 25, 2007, and completed Oct 1, 2007

Objective: To evaluate the biocompatibility of the LVRC system and its components

Background: The LVRD system consists of three implant sizes and a delivery system. The LVRD implants come in three different lengths; 70mm, 85mm, and 100mm. These implants are designed to be permanently placed into the airways of the lungs of patients suffering from emphysema. All three implant sizes are constructed with the same materials and undergo the same manufacturing processing. The LVRD Delivery System consists of 4 components; the introducer, the catheter, the cartridge, and forceps. These components are used to deploy and retrieve the LVRD Implants, if required, during the procedure and have limited patient contact. All components consist of materials that are commonly used for endovascular implants and delivery system products.

Methods: To determine what necessary biocompatibility tests needed to be performed on the Implant and the External Communicating Devices, Tables 1 & 2 of the FDA memorandum #G95-1 were referenced. The LVRD Implant is classified as a “tissue/bone” contacting Implant that has a “C-permanent (>30 days)” contact duration. The LVRD Delivery System is classified as External Communicating Devices with “tissue/bone/dentin communicating” body contact for the duration of “A-limited (24h)”. From these classifications, the memorandum outlines suggested biocompatibility tests that should be considered required or optional. The implant underwent all required biocompatibility tests except for Implantation (with Histology) and Carcinogenicity. Data and histology from PnuemRx’s 6 month GLP animal study (PRE-0015) will be supplemented in place of the implantation test. Carcinogenicity was not performed because the Implant currently consists of only one material, Nitinol, which is universally accepted as a highly biocompatible material and is commonly used in devices approved by the FDA and the EU for long term implant applications, such as Gore's commercially available EXCLUDER® AAA Endoprosthesis product. The tests that were performed are listed in Table 1 and completed test results are on file at PnuemRx, Inc. in the LVRD Design History File (DHF).

Results:

Table 1: Tests performed and final results

TESTS	RESULTS
Biocompatibility Tests for Implant	
1.1 Cytotoxicity: MEM Elution	Pass
1.2 Sensitization: Maximization	Pass
1.3 Irritation: Intracutaneous Reactivity	Pass
1.4 Systemic Toxicity: Systemic Injection	Pass
1.5 Systemic Toxicity: Material Mediated Pyrogen	Pass
1.6 Genotoxicity: Ames Test	Pass
1.7 Genotoxicity: Chromosomal Aberration	Pass
1.8 Genotoxicity: Mouse Lymphoma	Pass
Biocompatibility Tests for External Communicating Devices	
1.9 Cytotoxicity: MEM Elution	Pass
1.[illegible] Sensitization: Local Lymph Node Assay	Pass
1.[illegible] Irritation: Intracutaneous Reactivity	Pass

Conclusions: All of the tests that were conducted showed that the materials that are used in the LVRD are acceptably biocompatible as an Implant or External Communicating Device.

- **Retrieval Study (PRE-0014)**

Title: *Removal Evaluation of the Lung Volume Reduction Device (LVRD) Implants in the Bronchi in a Porcine Model.*

Initiated: April 10, 2007, and completed July 12, 2007

Objective: To evaluate our ability to explant LVRD devices after one and two month periods. The study entailed implantation of 3 LVRD prototype designs in the bronchi *in vivo* in the Yucatan Mini Swine. Specifically to evaluate:

- The ability of the operator / physician to recover the LVRDs after various amounts of time *in vivo*;
- The healing / tissue reaction at the implantation sites, characterized after various terms of implantation and post-removal healing;
- Any adverse events or findings associated with or attributed to the presence / removal of the LVRDs.
-

Results:

Device type	0 Months	1 Month	2 Months	3 Months
PneumRx LVRD Implant	N/A	N/A	8 implanted	8 attempted<br>8 removed<br>(1 mo *in-vivo*)
Straight Leg Prototype	16 implanted<br>4 attempted<br>4 removed<br>(0 mo *in-vivo*)	12 attempted<br>3 removed<br>(1 mo *in-vivo*)	9 attempted<br>1 removed<br>(2 mo *in-vivo*)	N/A
Curve Tail Prototype	N/A	N/A	N/A	6 implanted<br>4attempted<br>4 removed<br>(0 mo *in vivo*)

Conclusions:

- No intra-operative or post-operative adverse events associated with the implants or delivery system.
- All device designs were acutely removable.
- There was a 100% success rate for the PneumRx implant product after 1 month *in vivo.*
- PneumRx LVRD design was shown to be superior to the other prototypes tested.
- Histology:
  - The inflammation around the devices was shown to have a subsiding effect over time – i.e. the inflammatory response associated with device removal lessens over time
  - Observed fibrosis was considered normal and typical for any foreign body implant
  - Histological reactions observed in this study were attributed to multiple bronchoscopic evaluations and aggressive retrieval attempts used on prototype devices.
- The use of removal techniques (e.g. removing in-grown tissue with biopsy forceps), elicits a more severe histological reaction compared to simply leaving the device *in-situ.*

- **GLP Study (PRE-0015)**

Title: *A 3 Month and 6 Month In Vivo Sub Chronic and Chronic Evaluation Following Implantation of the PneumRx, Inc. Lung Volume Reduction Device in Porcine Lung.*

Initiated: March 28, 2007, interim report completed August 15 2007

Objective: To evaluate the *in vivo* 90 and 180-day safety of the LVRD implant and the immediate safety data on the LVRD catheter delivery system in a porcine model.

Results: Local response to the PneumRx LVRD implant, assessed by bronchoscopy and fluoroscopy, included complete shape recovery of test articles during the *in vivo* period and no migration of the LVRD Implant out of the treated airway. No clinically relevant systemic or regional responses to the LVRD implant occurred. One intra-operative adverse event was noted; a pneumothorax was caused by the interventionalist advancing the introducer into the pleural space during one procedure. The pneumothorax was resolved immediately (via chest tube), the animal was recovered and survived for the duration of the study. Gross and histological tissue evaluation confirmed the absence of subchronic or chronic toxicity. Histological evaluation of 33 sections obtained from the treated lungs of 3 study animals revealed a normal foreign body tissue response to the test article. No significant differences were apparent between the animals treated with the test articles or the sham control treatments.

Conclusion: The lack of local, systemic, and regional responses to the LVRD implant supports good overall device safety in the porcine model. All animals survived the 90-day evaluation period and no post-operative complications related to the test articles occurred. As a result of the pneumothorax the introducer was redesigned to include radio-opaque markers and an atraumatic “bumper” tip. LVRD delivery and deployment were rated as successful for all test articles. This study confirmed the *in vivo* 90-day safety of the LVRD implant and the immediate safety of the LVRD catheter delivery system in a porcine model.

- **Minimum Profile LVRD Retrieval Study (PRE0020)**

Title: Minimum Profile LVRD Study

Initiated: February 7, 2008 and completed on April 8, 2008

Objective:
To evaluate the moderate term safety and retrievability of the minimum profile prototype version on the PneumRx Lung Volume Reduction Device (LVRD) after two (2) months *in vivo*. This study evaluated the implantation of a total of sixteen (16) non-jacketed devices into two (2) Yucatan Mini Swine. A minimum of eight (8) devices were implanted in each swine; four (4) in each side of the lung. The right lungs contained devices with 0.05”-0.06” balls on the proximal ends and; the left lungs contained devices with a ball size of 0.3”-0.4”.
Specific evaluations in this study were to:

- Evaluate the short and moderate term results from implantation of minimum profile LVRD design.
- Evaluate the ability of the operator / physician to recover the LVRDs after two (2) months *in vivo*.
- Evaluate the healing / tissue reaction at the implantation sites, and subsequent removal. Characterized after two (2) month implantation.
- Observe any adverse events or findings associated with or attributed to the presence / removal of the LVRDs
- Evaluate the functionality of the LVRDs or the mode of delivery.

Results:
At two (2) months, 10 of 16 devices were retrieved. There was a 100% success rate when the proximal balls were observable and reachable. When the proximal ball was not accessible, removal was not attempted, 6 of the 10 devices were left *in situ* for histological analysis. 5 out of the 6 devices left *in situ* contained a ball size of 0.3” -0.4”. The implants with the smaller balls showed a significantly higher in-growth rate and therefore a significantly lower successful removal rate (3/8 removed with ~0.038” proximal and distal balls vs. 7/8 removed with ~0.055” proximal and distal balls.)

Test animals showed no clinical signs of acute or chronic distress due to the device implantation (i.e. no coughing, steady appetite, steady activity level, no infection, no pneumonia, etc.) Histology results showed similar tissue reactions as compared to previous animal studies, showing mild inflammation and fibrosis in the regions of the LVRD implants.

Conclusions:

- Due to the lower in-growth rate and higher retrieval rate the current ball size (~0.055”) should be maintained in the LVRD design.
- The non-jacketed “minimum profile design” LVRD had similar histological results in this study as to the previous studies. This demonstrates an equivalency between designs and the overall safety of the non-jacketed LVRD implants.
- Proceed with non-jacketed implants

- **First-in-Man Clinical Pilot Study**

Title: CLN0006.01.p.D **“**Evaluation of the PneumRx, Inc. Lung Volume Reduction Device for the Treatment of Emphysema”

Initiated: December, 2007

Current Status: Ongoing

Objective: The objective of this study is to demonstrate the safety of the PneumRx, Inc. Lung Volume Reduction Device (LVRD) in a population of patients with emphysema. This is an unblinded, single-arm, single site study. The study has been approved by the Freiburger Ethik-Kommission International and by the ethics committee at the University of Heidelberg.

Methods:
Patients with severe emphysema were treated in two bronchoscopy sessions – one session for each lung. In each session, LVRCs were placed into the lung area with the most severely damaged parenchyma. Patients were followed for 3 months following their last procedure. This protocol has been approved to enroll total of 15 subjects.

Specific evaluations in this study were to:
o Evaluate Adverse Events associated with implantation of Lung Volume Reduction Coils
o Evaluate the following outcome variables:
▪ St George’s Respiratory Questionnaire (SGRQ)
▪ Pulmonary Function Tests
▪ Pulmonary volumes, including Residual Volume (RV), Total Lung Capacity (TLC)
▪ 6 Minute Walk Test (6MWT)

Results:
To date, 9 patients have received at least one treatment and the data collected to date from the first 5 patients have been monitored. Each of the first 5 patients has had two bronchoscopic treatments and has received 5 LVRCs in each lung, except for the first treatment of the first patient, where only 4 LVRCs were implanted. All implantation procedures to date have progressed with no intra-procedural complications.

Safety: The analysis to date of the monitored patients:
- No deaths
- No pneumothorax in any patient
- No device migration or expectoration in any patient
- No pneumonia in any patient
- No coils required removal
- 3 patients experienced “Bronchoscopic COPD exacerbation” (transient moderate dyspnea and mild/moderate cough, hoarseness). Treated with standard medical therapy
- All Adverse Events resolved rapidly

Effectiveness: A summary of effectiveness measures, organized by emphysema type, is presented in the table below.

	**Change at 3-Month Follow-up**	
	**Heterogeneous N=3**	**Homogeneous N=2**
Change in SGRQ (± SEM)	-8 ± 9	-13 ± 5
% Change in FEV_1 (± SEM)	9% ± 9%	0.4% ± 7%
% Change in FVC (± SEM)	11% ± 10%	4% ± 5%
% Change in RV (± SEM)	-4% ± 19%	-6% ± 8%
% Change in RV/TLC (± SEM)	-4% ± 6%	-3% ± 2%
% Change in 6MWT (± SEM)	19% ± 8%	20% ±30%

Conclusions:

- The implantation of LVRCs appears to be safe.
- There have been no unanticipated adverse events to date.
- Mean change from baseline of all outcome variables has been in the direction of patient improvement.
- The study continues and is expected to close enrollment by the end of 2008.

3 Study Objectives

The objective of this study is to evaluate the safety and effectiveness of the PneumRx, Inc. LVRC in patients with heterogeneous and homogenous emphysema.

3.1 Primary efficacy endpoint:

- The primary efficacy endpoint will be the difference between treatment and control groups in the change in St. George's Respiratory Questionnaire (SGRQ) from Baseline (Pre-Treatment Visit) to the three month Follow-Up Visit.

3.2 Secondary efficacy endpoints:

The Secondary Endpoints will be the differences between Treatment and Control Groups in:

- Percent change in forced expiratory volume in 1 second (FEV_1) from Baseline (Pre-Treatment Visit) compared to one and three-month Follow-Up Visits

- Decrease in the Total Lung Capacity (TLC) and Residual Volume from Baseline (Pre-Treatment Visit) compared to one and three-month Follow-Up Visits.

- Decrease in the Residual Volume (RV) from Baseline (Pre-Treatment Visit) compared to one and three-month Follow-Up Visits.

- Improvement in the 6 minute walk test from Baseline (Pre-Treatment Visit) compared to one and three-month Follow-Up Visits.

- Improvement in the mMRC Dyspnea Scale from Baseline (Pre-Treatment Visit) compared to one and three-month Follow-Up Visits.

- Decrease in the need for supplemental O_2 from Baseline (Pre-Treatment Visit) compared to one and three-month Follow-Up Visits.

3.3 Safety Objectives:

The safety objective of this study is to identify the potential number and type of device-related and procedure-related adverse effects attributed to the use of PneumRx, Inc. LVRC.

3.4 Safety Analysis:

All adverse events reported will be listed, documenting course, outcome, severity, seriousness, possible relationship to the procedure, and possible relationship to the study device. Verbatim terms reported on case report forms will be mapped to standard Preferred Terms and System/Organ/Class using the MedDRA dictionary. A treatment-emergent adverse event will be defined as any adverse event that began during or after the initiation of study treatment, i.e. during or after the implant of the PneumRx, Inc. LVRC.

In summary tabulations for a given AE Preferred Term, counting will be performed by patient and not event, i.e. a patient reporting the same AE more than once will have that event counted only once, at the most severe and most-related occurrence. An adverse event that worsens in severity over time will be captured as multiple unique events, with the onset date of the new event corresponding to the date of worsening severity.

The number and percent of patients experiencing each Preferred Term will be summarized by site, severity, relationship to study treatment, seriousness. Furthermore, the number and percent of patients who withdrew or discontinued from the study due to an adverse event will be tabulated.

4 Study Design

4.1 Design Overview

This will be a multicenter, randomized, controlled, open label study comparing outcomes between the treatment and control groups. Subjects will be block randomized in a Treatment to Control ratio of 1:1.

4.2 Number of Subjects

The number of patients needed to demonstrate statistical significance ($\alpha<0.05$, $\beta=0.84$) of the difference in the proportion of patients reporting an improvement in SGRQ of 4 points or more is estimated to be 42. This is based on an estimated treatment effect of 0.6 (i.e., 60% treated patients report an SGRQ improvement > 4) and a change of 0.2 in the control group (i.e., 20% Control patients report an SGRQ change >4). Since we are testing the superiority of the treatment group a one-sided test is appropriate.

We will therefore recruit 45 patients into the study.

4.3 Treatment Group

The Treatment Group will have approximately 20 evaluable heterogeneous or homogenous emphysema patients. Each Treatment Group patient will be treated with the device with up to 2 bronchoscopies, separated by at least one month. They will be followed for 3 months after the final treatment.

4.4 Control Group

The Control Group will have approximately 20 evaluable patients who will be given the standard of care for patients with this condition. They will receive the same number of visits to study staff as subjects in the Treatment Group. Following 3 months of Control care, they will be treated with the test device and followed in the same manner as subjects in the Treatment Group.

4.5 Population

The population will include all patients who have met the inclusion/exclusion study criteria for the PneumRx, Inc. LVRC implant. Subjects enrolled in the study may or may not have had respiratory rehabilitation therapy prior to being enrolled in the study.

4.6 Demographic and Baseline Characteristics

Demographics and patient characteristics at baseline will be summarized to include age at enrollment, sex, and ethnic origin.

4.7 Safety Evaluation

Safety will be evaluated by collection of adverse events from entry into the study until exit, three months following the last LVRC implantation.

Following treatment, the phone interview and study visits, the patients will be instructed to report to the investigator any adverse physical or mental changes they experienced since the previous visit/interview. All such adverse events reported by the patients or observed by the investigators will be recorded.

4.8 Brief Description of Study

The complete Study and its required visits and assessments will be carefully discussed with the study patients using an Institutional Review Board (IRB) or an Ethics Committee (EC) approved Informed Consent. The Informed Consent will contain all essential elements including a description of the research, expected duration and procedures, statement of the patient’s right to decline to participate or to withdraw from the study at any time and for any reason without fear of retribution, potential risks, discomforts or adverse effects, prospective benefits, limits of confidentiality, incentive for participation, timely dissemination of any new information that becomes available, and contact information of the research personnel. All patients will sign an Informed Consent prior to any procedures being performed to evaluate their eligibility for participation in the Study. A Sample Informed Consent is provided in Appendix B.

A physician who feels the patient may benefit from this study will refer the patient to the study investigator. If the patient is willing to participate (s)he will sign the Informed Consent. Once the Informed Consent is signed the patient will go through an initial screening evaluation. During the screening visit, demographic information, medical history, physical exam, smoking history and questionnaires will be collected to ensure the patient meets inclusion and exclusion criteria. If the patient does not meet the inclusion / exclusion criteria (s)he will be excluded from the study.

If the patient has met the initial inclusion / exclusion criteria (s)he will continue the screening evaluation to ensure (s)he meets the other inclusion / exclusion criteria. The investigator will perform other tests such as spirometry and body plethysmography, CT scans, X-rays as standard care, walk tests, blood tests, etc.

Once the patient has completed the pre-treatment tests and meets the remaining inclusion / exclusion criteria, the patient will be randomized to either the Treatment Group or the Control Group.

Treatment Group
Subjects randomized to the Treatment Group will undergo bronchoscopy with LVRC implants according to the Instructions for Use in the Investigator’s Brochure. Once treated with the device the patient will remain in the hospital under observation for one day. Following hospital discharge, the patient will be contacted by phone one week after implant and will be seen in the department at one month. After the one month visit the patient may be scheduled for more implants. If the patient is not scheduled for additional implants the patient will be seen at three months, six months, and twelve months (See Table 1). After the three month visit, the patient will be exited from the study.

If the patient is treated with the device a second time, the patient will remain in the hospital under observation for one day. Following hospital discharge, the patient will be contacted by phone one week after implant and will be seen one month, three months, six months, and twelve months after implant (See Table 2). After the twelve month visit, the patient will be exited from the study.

Control Group

Subjects randomized to the Control Group will not immediately undergo bronchoscopy, but will attend visits with the study staff as follows. One week after randomization they will have a “treatment #1” visit, during which they will have a limited physical exam and will be queried about adverse events. They will then have a telephone call at 1 week post-“treatment” 1, and a follow-up visit at 1 month post-“treatment” 1. After the 1-month visit, Control Group patients will have a “Treatment #2” visit, at which they will have the same testing as at the “Treatment #1 visit. Following “Treatment”#2 visit, patients will have a telephone call at 1 month and follow-up visits at 1 and 3 months.

Following the 3-month post-“Treatment #2” visit, patients in the Control Group will undergo bronchoscopy and LVRC implants following the same protocol as patients in the Treatment Group. Control Group patients will not be required to repeat all the tests done at Visit 1, Pretreatment.

Testing and procedures for the Treatment and Control Groups is presented in Tables 1 and 2, respectively.

Table 1. Study Procedures for Treatment Group

Procedure / Assessment	Visit 1 Pre-Treatment	Visit 2 Bronchoscopy #1 (Day 1 – Day 2)	Visit 3 F/U (1 Week post bronchoscopy #1) Phone Call	Visit 4 F/U (1 Month post bronchoscopy #1)	Visit 5 Bronchoscopy #2 (Day 1 – Day 2)	Visit 6 F/U (1 Week post bronchoscopy#2) Phone Call	Visit 7 F/U (1 Month post bronchoscopy #2)	Visit 8 F/U (3 Months post bronchoscopy #2)	Visit 9 F/U (6 Months post bronchoscopy #2)	Visit 10 F/U (12 Months post bronchoscopy #2)
Informed Consent	X									
Medical History	X									
Physical Examination to include SpO_2	X	X		X	X		X	X	X	X
QOL (St. Georges)	X			X			X	X	X	X
Spirometry	X			X			X	X	X	X
Lung Volumes	X			X			X	X	X	X
Hematology, Coagulation, Blood Chemistry	X									
Blood Gases	X									
EKG	X									
Echocardiogram	X									
Dyspnea Scale mMRC	X			X			X	X	X	X
6 Minute Walk Test	X			X			X	X	X	X
Concomitant Medication / O_2 Use	X	X	X	X	X	X	X	X	X	X
Pregnancy Testing	X	X		X	X		X	X	X	X
High Resolution CT Scan(Standard care)	X							X	X	
Chest X-Ray Standard Care	X									
Bronchoscopy / LVRC Placement		X			X					
Review Patient Status	X	X	X	X	X	X	X	X	X	X
Exit Study								X	X	X

Table 2. Study Procedures for Control Group (First Phase)

Procedure / Assessment	Visit 1 Pre-Treatment	Visit 2 "Treatment" #1 (Day 1 – Day 2)	Visit 3 F/U (1 Week) Phone Call	Visit 4 F/U (1 Month)	Visit 5 "Treatment" #2 (Day 1 – Day 2)	Visit 6 F/U (1 Week post-"treatment" #2) Phone Call	Visit 7 F/U (1 Month post-"treatment" #2)	Visit 8 F/U (3 Months post-"treatment" #2)
Informed Consent	X							
Medical History	X							
Physical Examination to include SpO_2	X	X		X	X		X	X
QOL (St. Georges)	X			X			X	X
Spirometry	X			X			X	X
Lung Volumes	X			X			X	X
Hematology, Coagulation, Blood Chemistry	X							
Blood Gases	X							
EKG	X							
Echocardiogram	X							
Dyspnea Scale mMRC	X			X			X	X
6 Minute Walk Test	X			X			X	X
Concomitant Medication / O_2 Use	X	X	X	X	X	X	X	X
Pregnancy Testing								
High Resolution CT Scan(as standard Care)	X							
Chest X-Ray (as standard care)	X							
Bronchoscopy / LVRC Placement								
Review Patient Status	X	X	X	X	X	X	X	X

5 Study Subject Recruitment

All study patients will be volunteers who meet the inclusion / exclusion criteria including a willingness to read, understand, and sign the Informed Consent. Recruitment of study patients will likely be from the pool of patients attending clinics at the study site. Referrals may be sought from local physicians/general practitioners in the community who see and treat emphysema patients.

The duration of study recruitment period is expected to last between 1 and 6 months.

5.1 Inclusion Criteria

Patients must meet **all** of the following inclusion criteria to be entered into the study:

1. Patient $\geq$ 35 years of age
2. High Resolution CT scan indicates unilateral or bilateral emphysema
3. High Resolution CT scan indicates homogeneous or heterogeneous emphysema
4. Patient has post- bronchodilator FEV_1 less than or equal to 45% predicted
5. Total Lung Capacity > 100% predicted
6. Patient has marked dyspnea scoring $\geq$2 on mMRC scale of 0-4
7. Patient has stopped smoking for a minimum of 8 weeks prior to entering the study
8. Patient (and legal guardian if applicable) read, understood and signed the Informed Consent form

5.2 Exclusion Criteria

Patients will be excluded from the study if **any** of the following conditions apply:

1. Patient has a change in FEV_1 > 20% post-bronchodilator.
2. Patients DLCO < 20% predicted
3. Patient has a history of recurrent clinically significant respiratory infection
4. Patient has uncontrolled pulmonary hypertension defined by right ventricular pressure >50mmHg and/or evidenced by echocardiogram
5. Patient has an inability to walk >140 meters (150 yards) in 6 minutes
6. Patient has evidence of other disease that may compromise survival such as lung cancer, renal failure, etc
7. Patient is pregnant or lactating
8. Patient has an inability to tolerate bronchoscopy under heavy sedation or anesthesia
9. Patient has clinically significant bronchiectasis
10. Patient has giant bullae > 1/3 lung volume
11. Patient has had previous LVR surgery, lung transplant or lobectomy
12. Patient has been involved in other pulmonary drug studies with 30 days prior to this study
13. Patient is taking > 20mg prednisone (or similar steroid) daily
14. Patient is on Plavix or has not been weaned off prior to procedure
15. Patient has other disease that would interfere with completion of study, follow up assessments or that would adversely affect outcomes

6 Study Plan

6.1 Test Device

The device being evaluated in this clinical study is the PneumRx, Inc. LVRC

Investigators will be fully trained in the proper use and operation of the PneumRx, Inc. LVRC before initiation of any treatment. Training will include hands-on use of the system and didactic sessions. In addition, on-site training will be provided to the investigator, co-investigators and other study support personnel before the first treatment at the study site. If necessary, additional training will be provided. PneumRx, Inc. personnel will be available to provide any additional technical support during treatment sessions until the investigator and their team feels comfortable with the use of the device.

6.2 Detailed Study Description

6.2.1 Informed Consent

- Obtain a signed Informed Consent from the patient prior to beginning the initial screening.
- Provide the patient with a copy of the signed Informed Consent for their records.

6.2.2 Study Identification Number

- Assign the patient a unique study identification number (Study ID number) at the time of signing an Informed Consent.

6.2.3 Screening Period

Tests are performed during the screening period to determine the patient’s eligibility for the study.

6.2.4 Screening Evaluations

Perform the following evaluations during the screening period:

- Detailed medical history, to include the number of years the patient has been diagnosed with emphysema, other significant illnesses, current smoking status and history, medications, O_2 use etc
- Physical examination, including SpO_2 and breath sounds
- Resting Electrocardiogram (EKG)
- Echocardiogram
- Blood tests to include Hemoglobin, Haematocrit, White Blood Cells (WBC), Platelet count, Prothrombin Time (PT, Sodium, Potassium, Chloride, Glucose, Total Protein, Albumin, Urea Nitrogen (BUN), and Creatinine
- Pregnancy test for females of child bearing potential
- Arterial Blood Gasses – these will be taken from the ear lobe

- Pre and Post-bronchodilator spirometry
- Lung volume measurements using body plethysmography
- High Resolution CT Scan (as standard care for diagnosis)
- mMRC dyspnea scale
- 6 minute walk test.
-

6.2.5 Randomization

- If the patient meets all entry criteria open the next sequential Randomization Assignment envelope supplied by the sponsor and inform the patient of his/her assignment.
- Randomization assignment determines the next step:
- Patients assigned to the Control Group will proceed to "Treatment #1" step 6.2.6 and complete all steps through step 0 .
- Patients assigned to the Treatment Group will proceed directly to Bronchoscopy Step 6.2.14.

6.2.6 "Treatment #1" **(Control Patients Only)**

- Record Adverse Events (See Section 7.0).
- Review Medications.
- Review Patient Status.

6.2.7 1 Week Post "Treatment #1" Follow Up Phone Call

- Contact Patient via telephone 1 week after "Treatment #1" to assess status.
- Review medications and O_2 use.
- Record Adverse Events (See Section 7.0).

6.2.8 1 Month Post "Treatment #1" Follow Up Evaluation

- Perform focused physical exam including Vital Signs (heart rate, blood pressure, temperature, respiratory rate, SpO_2) and breath sounds.
- Perform Pre- and Post-bronchodilator spirometry.
- Lung volume measurements using body plethysmography
- Review medications and O_2 use.
- Administer St. George's Quality of Life Questionnaire.
- Administer mMRC Dyspnea Scale.
- Perform the 6 minute walk test.
- Record Adverse Events since last follow-up (See Section 7.0).

6.2.9 Post 1 Month Follow Up

- Following the successful completion of the 1 Month Follow Up Evaluation the patient can be scheduled his/her "Treatment #2" visit.

6.2.10 "Treatment #2" **(Control Patients Only)**

- Record Adverse Events (See Section 7.0).
- Review Medications.
- Review Patient Status.

6.2.11 1 Week Post "Treatment #2" Follow Up Phone Call

- Contact Patient via telephone 1 week after "Treatment #2" to assess status.
- Review medications and O_2 use.
- Record Adverse Events (See Section 7.0).

6.2.12 1 Month Post "Treatment #2" Follow Up Evaluation

- Perform focused physical exam including Vital Signs (heart rate, blood pressure, temperature, respiratory rate, SpO_2) and breath sounds.
- Perform Pre- and Post-bronchodilator spirometry.
- Lung volume measurements using body plethysmography
- Review medications and O_2 use.
- Administer St. George's Quality of Life Questionnaire.
- Administer mMRC Dyspnea Scale.
- Perform the 6 minute walk test.
- Record Adverse Events since last follow-up (See Section 7.0).

6.2.13 3 Month Post-"Treatment#2" Follow Up Evaluation

- Perform focused physical exam including Vital Signs (heart rate, blood pressure, temperature, respiratory rate, SpO_2) and breath sounds.
- Perform Pre and Post-bronchodilator spirometry.
- Lung volume measurements using body plethysmography.
- Review medications and O_2 use.
- Administer St. George's Quality of Life Questionnaire.
- Administer mMRC Dyspnea Scale.
- Perform the 6 minute walk test.
- Record Adverse Events since last follow-up (See Section 7.0).

6.2.14 Bronchoscopy #1 / LVRC Placement

- Perform pregnancy test for females of child bearing potential.
- Prescribe prednisolone 30 mg/day for 5 days, to start 3 days before bronchoscopy.
- Prepare patient for bronchoscopy per standard hospital practice.
- Begin prophylactic antibiotic treatment per standard hospital practice.
- Administer heavy sedation to perform LVRC placement.
- Insert the bronchoscope into the patient per manufacturer's instructions.
- Navigate the bronchoscope and identify the airways leading to the diseased parenchyma via fluoroscopy.
- Insert the PneumRx catheter/introducer into the working channel of the bronchoscope and deliver the device per the PneumRx instructions for use.
- Navigate the catheter/introducer to the distal airways and verify the position via fluoroscopy.
- Deliver the implant into the catheter and deploy the implant while monitoring the position via fluoroscopy.
- Only place the devices unilaterally. DO NOT place the devices in both the right and left lungs of the patient.
- Allow the subject to recover from sedation and monitor as per standard hospital practice.

6.2.15 Post Bronchoscopy Monitoring and Evaluations

- Patient will be monitored per standard hospital practice.
- Record Adverse Events (See Section 7.0).
- Admit patient to hospital for 1 day of observation per standard practice.

6.2.16 1 Day Hospital Stay

- Patient will be monitored daily per standard hospital practice.

6.2.17 1 Week Post-Bronchoscopy #1 Follow Up Phone Call

- Contact Patient via telephone 1 week after bronchoscopy session / LVRC procedure to assess status.
- Review medications and O_2 use.
- Record Adverse Events (See Section 7.0).

6.2.18 1 Month Post-Bronchoscopy #1 Follow Up Evaluation

- Focused physical exam including Vital Signs (heart rate, blood pressure, temperature, respiratory rate, SpO_2) and breath sounds.
- Perform Pre- and Post-bronchodilator spirometry.
- Lung volume measurements using body plethysmography.
- Review medications and O_2 use.
- Administer St. George's Quality of Life Questionnaire.
- Administer mMRC Dyspnea Scale.
- Perform the 6 minute walk test.
- Record Adverse Events since last follow-up (See Section 7.0).

6.2.19 Post 1 Month Follow Up Second Treatment Option

- Following the successful completion of the 1 Month Follow Up Evaluation the patient can be scheduled for additional LVRC placement. If the investigator feels that the patient may benefit with additional devices then the subject may be scheduled for another LVRC placement. If the patient does not have a second bronchoscopy, then proceed directly to 3-month follow-up Step 6.2.25 .

- During the second LVRC implantation procedure, the investigator may treat the same lung that was treated in the first procedure or may treat the contra-lateral lung.

6.2.20 Bronchoscopy #2 / LVRC Placement

- Perform pregnancy test for females of child bearing potential.
- Prescribe prednisolone 30 mg/day for 5 days, to start 3 days before bronchoscopy.
- Prepare patient for bronchoscopy per standard hospital practice.
- Begin prophylactic antibiotic treatment per standard hospital practice.
- Administer heavy sedation to perform LVRC placement.
- Insert the bronchoscope into the patient per manufacturer's instructions.
- Navigate the bronchoscope and identify the airways leading to the diseased parenchyma via fluoroscopy.
- Insert the PneumRx catheter/introducer into the working channel of the bronchoscope and deliver the device per the PneumRx instructions for use.
- Navigate the catheter/introducer to the distal airways and verify the position via fluoroscopy.
- Deliver the implant into the catheter and deploy the implant while monitoring the position via fluoroscopy.

- Only place the devices unilaterally. DO NOT place the devices in both the right and left lungs of the patient.
- Allow the subject to recover from sedation and monitor as per standard hospital practice.

6.2.21 Post Bronchoscopy Monitoring and Evaluations

- Patient will be monitored per standard hospital practice.
- Record Adverse Events (See Section 7.0).
- Admit patient to hospital for 1 day for observation per standard practice.

6.2.22 1 Day Hospital Stay

- Patient will be monitored daily per standard hospital practice.

6.2.23 1 Week Post Bronchoscopy #2 Follow Up Phone Call

- Contact Patient via telephone 1 week after bronchoscopy session / LVRC procedure to assess status.
- Review medications and O_2 use.
- Record Adverse Events (See Section 7.0).

6.2.24 1 Month post-bronchoscopy #2 Follow Up Evaluation

- Perform focused physical exam including Vital Signs (heart rate, blood pressure, temperature, respiratory rate, SpO_2) and breath sounds.
- Perform Pre- and Post-bronchodilator spirometry.
- Lung volume measurements using body plethysmography.
- Review medications and O_2 use.
- Administer St. George's Quality of Life Questionnaire.
- Administer mMRC Dyspnea Scale.
- Perform the 6 minute walk test.
- Record Adverse Events since last follow-up (See Section 7.0).

6.2.25 3 Month Post-Bronchoscopy #2 Follow Up Evaluation

- Perform focused physical exam including Vital Signs (heart rate, blood pressure, temperature, respiratory rate, SpO_2) and breath sounds.
- Perform Pre and Post-bronchodilator spirometry.
- Lung volume measurements using body plethysmography.
- Review medications and O_2 use.

- Perform High Resolution CT Scan (as standard care)
- Administer St. George's Quality of Life Questionnaire.
- Administer mMRC Dyspnea Scale.
- Perform the 6 minute walk test.
- Record Adverse Events since last follow-up (See Section 7.0).

6.2.26 3 Month Post-Bronchoscopy #2 Follow Up Evaluation

- Perform focused physical exam including Vital Signs (heart rate, blood pressure, temperature, respiratory rate, SpO_2) and breath sounds.
- Perform Pre and Post-bronchodilator spirometry.
- Lung volume measurement, Lung volumes will be measured using body plethysmography.
- Review medications and O_2 use.
- Perform High Resolution CT Scan. (As standard care)
- Administer St. George's Quality of Life Questionnaire.
- Administer mMRC Dyspnea Scale.
- Perform the 6 minute walk test.
- Record Adverse Events since last follow-up (See Section 7.0).

6.2.27 12 Month Post-Bronchoscopy #2 Follow Up Evaluation

- Perform focused physical exam including Vital Signs (heart rate, blood pressure, temperature, respiratory rate, SpO_2) and breath sounds.
- Perform Pre and Post-bronchodilator spirometry.
- Lung volume measurement, Lung volumes will be measured using body plethysmography.
- Review medications and O_2 use.
- Administer St. George's Quality of Life Questionnaire.
- Administer mMRC Dyspnea Scale.
- Perform the 6 minute walk test.
- Record Adverse Events since last follow-up (See Section 7.0).

6.2.28 Study Exit

- After successful completion of the 12 month post bronchoscopy visit the patient can be exited from the study.

7 Adverse Events

An adverse event is any sign, symptom, illness, clinically significant abnormal laboratory value, or other adverse medical event that appears or worsens in a patient during a clinical study, regardless of whether or not it is considered related to the procedure / device used as part of the protocol.

It is the responsibility of the investigator to report when an adverse event has occurred. Adverse event information will be collected throughout the study. Adverse events will be recorded on the Adverse Event CRF by the investigator or study coordinator. Event, date of onset, severity, duration, and relationship to the procedure will be recorded on the appropriate CRF. All adverse events will be monitored until they are adequately resolved or stabilized with no further significant change expected.

All serious adverse events must be reported to the trial monitor immediately or within 24 hours (one working day) by telephone (650-625 8910), FAX (650-625 8915), or E-mail barriew@pneumrx.com and to EU reg. Rep MPS Tel 0171 3594254, Fax 06442 962074 G. Froemel@mps-gmbh.eu To maintain patient confidentiality, the patient will only be identified by the patient number used on the case report forms. A written report must follow within five working days and is to include a full description of the event and sequelae, in the format detailed by the serious adverse event reporting form.

For all serious adverse events, reports relating to the patients subsequent medical follow-up must be submitted to PneumRx, Inc. regularly until the event has subsided or, in case of permanent impairment, until the event stabilizes and the overall clinical outcome has been ascertained.

Severity of Adverse Events: The following general definitions for rating severity of adverse events should be used for this study:

1. **Mild:** Awareness of signs or symptoms, but easily tolerated and transient; causing no loss of time from normal activities; symptoms would not require medication or a medical treatment; signs and symptoms are transient.

2. **Moderate:** Marked symptoms and discomfort severe enough to cause moderate interference with the patient's usual activities. Symptomatic treatment is possible.

3. **Severe:** Incapacitating with inability to do work or usual activities; signs and symptoms may be of systemic nature or require medical intervention and/or treatment. Hospitalization may be required or prolonged.

Serious Adverse Events (SAE): All adverse events are categorized as Mild, Moderate or Severe. Additionally, if an adverse event meets any of the following criteria, it is also categorized as a Serious Adverse Event (SAE) when the event is:

- Fatal
- Requires or prolongs hospitalization
- Causes substantial risk of dying at the time of the event (i.e., life-threatening)

- Results in permanent impairment of a body function or permanent damage to a body structure, or
- Requires medical or surgical intervention to prevent permanent impairment of a body function or permanent damage to a body structure

Note: This categorization for adverse events is independent of the severity rating applied above.

Unanticipated Adverse Device Effect (UADE): Any serious adverse effect on health or safety or any life-threatening problem or death caused by, or associated with, a device, if that effect, problem, or death was not previously identified in nature, severity, or degree of incidence in the investigational plan or application (including a supplementary plan or application), or any other unanticipated serious problem associated with a device that relates to the rights, safety, or welfare of subjects.

Adverse Event Relationship: The relationship of an Adverse Event to the underlying disease or to the procedure will be attributed using the following definitions:

1. **Not Related:** There is no evidence that the adverse event has a relationship to the procedure performed.

2. **Possibly Related**: The adverse event has a timely relationship to procedure performed. However, a potential alternative etiology may be responsible for the adverse event.

3. **Probably Related**: The adverse event has a timely relationship to study procedure performed and the causative relationship can clearly be established. No potential alternative etiology is apparent.

8 Risks and Benefits

8.1 Potential Risks to the Patient

Participation in this clinical study may expose the patient to the following potential risks associated with the device or the procedure:

1. Bronchoscopy

With any bronchoscopic procedure, there is the possibility of fever, bleeding, laryngospasm, bronchospasm, irregular heartbeat, shortness of breath, infection, transient infiltrates, pneumonia (Djukanovic, 1998), pneumothorax (Bleeker, 1992) or syncope. In the event that any of these were to occur, the Subject will be treated for the condition. Some subjects may experience wheezing, coughing, or shortness of breath during the first few days following a bronchoscopy procedure.

2. Pneumonia or infection

There is a risk of developing pneumonia as a result of excess mucus production or because of other theoretical reasons such as impairment of the ability for the lung to clear mucus.

3. Haemoptysis (coughing up blood > 5ml)

4. Conscious Sedation/Anesthesia

There is a potential risk of developing side effects associated with the use of anesthesia. The risks of anesthesia depend on the agents and/or gases used. The risks of anesthesia include postoperative pain, nausea and vomiting, dizziness, drowsiness, shivering, liver toxicity and/or cardiovascular events.

Trained professionals with extensive experience and expertise who routinely administer local anesthesia with conscious sedation to patients requiring multiple procedures will be responsible for the induction and associated monitoring required for this study. In addition, study patients will undergo extensive monitoring throughout the recovery period as well after the recovery period, as indicated.

The following are potential risks that are associated with the device:

5. Pneumothorax (presence of air within the pleural space)

6. Unanticipated reaction to the study device that could require emergency intervention to remove the study device

The following are potential risks that are associated with the tests required as part of the study conduct:

7. Blood draws

The risks of blood draws include temporary pain and discomfort from the needle stick, and/or tenderness, redness or bruising at the site, bleeding, fainting and lightheadedness.

8. Pulmonary function tests

Pulmonary function tests are low risk procedures. They may occasionally cause dizziness and/or slight chest discomfort due to muscle soreness, but these are self-limited.

9. Chest X-rays CT Scans and Fluoroscopy

Study patients will have radiation exposure as a result of the chest X-rays, CT scans and fluoroscopy as required. The doses of radiation used typically are so small that the risk of these procedures is difficult to measure.

The following risks are associated with the use of certain drugs that are required as part of the study conduct:

10. Medications required to perform bronchoscopy

Drugs required for bronchoscopy could include lidocaine, atropine and one of the benzodiazepines. Although these drugs each have a number of potentially significant side effects, they are commonly used to perform bronchoscopy (Djukanovic, 1998).

Lidocaine toxicity has been described in association with bronchoscopy. At least one death has been reported in the literature as a result of lidocaine toxicity in a research Subject who underwent bronchoscopy (Clinical Trials Advisory Newsletter, 1996). Amounts of lidocaine given will be monitored and recorded. Conscious sedation can be associated with respiratory suppression resulting in hypoxemia and the need for increased supplemental oxygen or the need for intubation with mechanical ventilation. In addition, sedation can result in cardiovascular compromise with hypotension. To minimize these complications, sedation will be given in accordance with conscious sedation protocols applicable at the participating hospital and administered by trained professionals with experience in conscious sedation and ventilation.

Patients with known sensitivity to drugs required to perform bronchoscopy are excluded from study participation. Should a patient experience a significant side effect for which there is concern, they will be managed as appropriate.

Insurance Coverage

If an incident occurs, an insurance policy has been taken out to cover damages within the legally prescribed scope. The insured amount is € 500,000.

8.2 Potential Benefits to the Patient

It is possible that Patient will not receive any benefits from treatment with the PneumRx, Inc. LVRC.

Potential benefits of the PneumRx, Inc. LVRC treatment that may be realized by study participants include, overall fewer symptoms related to emphysema, and improved quality of life.

One potential benefit to patients participating in the study is the ability to learn more about their emphysema, based on the assessments that will be performed throughout the course of the study. In addition, the pateints will receive education about the monitoring of their disease, as will be required for study participation.

PneumRx, Inc. the study sponsor will pay all medical costs over the usual costs of treatment associated with Study participation or standard medical care.

The results of this study may help other emphysema patients to gain access to a device that may improve their quality of life and general health.

9 Study Monitoring

PneumRx, Inc. or its designee will periodically review the data to ensure that the study investigator is in compliance with the protocol and the investigator's agreement. PneumRx, Inc. or its designee will monitor the sites to ensure that the completed CRFs match the medical records and to resolve any discrepancies.

PneumRx, Inc. or its designee will meet with the investigator prior to the initiation of the study in order to review the adequacy of the patient population, facilities, and equipment with respect to the needs of the study, and to familiarize the investigator with the study protocol.

PneumRx, Inc. or its designee will meet with the investigator at the time enrollment is initiated in order to ensure that patients are being properly selected, that the methods described in the study protocol are thoroughly understood by the investigator, and that study data are being correctly recorded.

PneumRx, Inc. or its designee will visit the clinical site periodically during the course of the study to review completed CRFs and monitor the reported data to source documentation. Additionally, telephone consultation will occur as necessary during the course of the study to ensure the proper progress and documentation of the study findings.

10 Responsibilities of the Sponsor

The sponsor of this clinical trial is PneumRx, Inc. of Mountain View, CA, U.S.A. The sponsor is committed to:

- Conducting this clinical trial in compliance with Good Clinical Practices (GCP) Guidelines.

- Protecting the rights, health, safety and welfare of study patients; the sponsor is responsible for obtaining and reviewing copies of research ethics board approvals and will verify that appropriate patient Informed Consent is obtained.

- Informing the clinical investigator of any new information about the study that may affect the health, safety or welfare of the patients, or which may influence their decision to continue participating in the study.

- Providing the clinical investigator with the study protocol the CRFs on which to document the study evaluation variables for each patient entered into the Study.

- Providing the statistical analysis and study report-writing resources necessary to complete reporting of the study results.

- Ensuring proper investigative site monitoring.

- Selecting qualified investigators to conduct this clinical trial.

- Maintaining copies of correspondence, records of shipment and disposition of devices, adverse device effects, records related to the signed investigator agreements, and other records related to the clinical study.

11 Responsibilities of the Principal Investigator

The Principal Investigator (PI) participating in this clinical trial must be a licensed physician in his/her country of employment. The investigator will affirm by his/her signature on the Investigator's Agreement that he/she will fulfill his/her responsibilities relative to this clinical trial.

- Patient Selection

The investigator is responsible for ensuring that all patients entering the study conform to the patient inclusion criteria and that no exclusion criteria apply.

- Ethics Approval

The investigator is responsible for obtaining ethics approval from the institution at which he or she shall perform the procedure, prior to enrolling any patients in the study. The Informed Consent document to be used will also be submitted by the Investigator to the ethics committee for approval prior to initiation of the study. The investigator is also responsible for providing any other additional documentation relevant to the study as required by ethics for complete review of the study. Written assurance of ethics approval of the trial plan and the Informed Consent document must be provided to the sponsor prior to initiation of the study.

- Informed Consent

The investigator is responsible for fully discussing the nature of the study, the possible risks, and the alternative treatments with prospective patients prior to their enrollment in the study. The investigator is responsible for obtaining written Informed Consent from each patient prior to enrollment in the trial. The Informed Consent form to be used should be that version of the document approved by ethics. The signed Informed Consent form will be maintained in the patient’s medical record, and a copy of the signed Informed Consent form will become an integral part of each case report file retained by the Investigator. A copy of the signed Informed Consent form shall also be given to the patient who signed the form.

A copy of the proposed Informed Consent form for this clinical trial is provided as an attachment to this document.

- Patient Evaluations and Data Reporting

The investigator is responsible for performing the patient evaluations as described in this trial plan. Regulations require that the study investigator maintain information in the study patient’s medical records (i.e. source documentation) to corroborate data collected on the case report forms (CRFs).

All information generated by the patient evaluations is to be transferred from the source documentation and recorded onto the CRFs provided by the sponsor. Paper CRFs, should be filled out in blue or black ink, or should be typewritten. Any necessary corrections should be made by a single strikethrough in ink, initialed and dated by study site personnel. Correction fluid may not be used. The investigator will review, correct as needed, and sign off on the accuracy and completeness of the CRF data entered on the forms. Patient casebooks may be printed for review by authorized regulatory bodies. Original laboratory reports are to be retained by the Investigator, and the resulting data shall be entered onto the appropriate CRFs.

The sponsor will routinely monitor the patient CRFs on an ongoing basis.

The investigator is also responsible for submitting reports to PneumRx, Inc. and the reviewing ethics as specified in this protocol.

- Protocol Deviations

The study investigator should not deviate from this protocol, unless the trial plan poses unacceptable risks to the health or welfare of the involved individual patient.

The investigator shall notify PneumRx Inc. and the reviewing ethics committee of any deviation from the protocol intended to protect the life or physical well being of a patient in an emergency. Such notice shall be given as soon as possible, but in no event later than five working days after the emergency occurred. Except in such an emergency, prior approval of PneumRx Inc. is required for any deviation from the protocol. Approval from the ethics committee also is required if these changes or deviations are expected to affect the rights, safety or welfare of human patients.

- Record Retention

The investigator shall maintain all original records as required by local regulation or law. PneumRx Inc. will provide record retention dates to all investigators

- Investigational Device Accountability

The investigator must maintain accurate records of the receipt of all investigational devices shipped by the sponsor, including the date and lot numbers of devices received. In addition, accurate records must be kept regarding the date and quantities of investigational devices received, dispensed and returned. Information regarding the specific identification numbers for investigation devices used is to be recorded onto the appropriate device accountability log for each patient undergoing the treatment procedure throughout the course of the study. The Investigator must assure that study supplies are dispensed only to patients properly enrolled in the study and under the direct supervision of the investigator or co-investigators.

All used and unused investigational supplies, as well as all labeled containers, are to be returned to the sponsor as soon as practical upon request by the sponsor or designee or upon completion of the study. Investigational material accounting procedures must be completed before the study is considered terminated.

12 Good Clinical Practice & Regulatory Requirements

Informed Consent
Written Informed Consent for the study must be obtained from all patients who will participate in this clinical trial prior to their participation.

A draft of the Informed Consent form that can be used for this study is provided as an Appendix to this document. Individual institutions may revise the form with information that would meaningfully add to the protection of the rights and welfare of patients. Prior to submitting the revised Informed Consent form to the IRB/EC for review, the investigator is to receive authorization of the revisions by PneumRx Inc. clinical affairs staff. Then, the EC at each clinical site will approve the consent form prior to study initiation. The investigator at each institution shall submit the EC approved consent form to the sponsor who shall review the form to ensure compliance with applicable regulations.

IRB/EC Approval
This Study may not be initiated at any site until the IRB/EC has reviewed the study protocol and the Informed Consent documents, and has provided written approval prior to initiation of the Study.

Patient Confidentiality
Patient confidentiality shall be maintained at all times throughout the conduct of this trial, and all patient data shall be maintained secure against unauthorized access. Possible review and photocopying of the patient's records by regulatory authorities could occur. In addition the sponsor of this study, PneumRx Inc. and its representatives may review and photocopy the patient's records. Copies (electronic or hard copy) of the patient's CT Scans will be collected as study data. In the event patient's data are used for educational, presentation, and/or publication purposes, patient identity will be masked to protect the patient's confidentiality.

13 References

1. Bleeker ER, ER McFadden, et al. Editorial - Investigative bronchoscopy in subjects with asthma and other obstructive pulmonary diseases, whether and when. Chest 1992; 101(2): 297-298.

2. Clinical Trials Advisory Newsletter, Death of a healthy college student volunteer in a research study: special report. Naples, FL: Global Success Corporation, 1996.

3. Djukanovic R, R Dahl, et al. Safety of biopsies and bronchoalveolar lavage. Eur Respir J 1998; 11: Suppl 26: 39s-41s.

4. Respiratory Physiology, The Essentials, JB West. Lippencott, Williams, Wilcons; Philadelphia, PA. 6th Edition, 2000.

5. World Medical Association Declaration of Helsinki, as most recently amended by the 52nd Annual WMA General Assembly, Edinburgh, Scotland, October 2000, and the note of clarification on Paragraph 29 added by WMA General Assembly, Washington, DC, 2002 and the note of clarification on Paragraph 30 added by WMA General Assembly, Tokyo, 2004
